# Histone demethylase Kdm5c regulates osteogenesis and bone formation via PI3K/Akt/HIF1α and Wnt/β-catenin signaling pathways

**DOI:** 10.1016/j.gendis.2023.02.041

**Published:** 2023-04-04

**Authors:** Ying Li, Lingli Ding, Yage Zhang, Bingyu Zhou, Gene Chi Wai Man, Min Wang, Jinglan Li, Yamei Liu, Weiping Lin, Haibin Wang, Sien Lin, Liangliang Xu

**Affiliations:** a Key Laboratory of Orthopaedics & Traumatology, Lingnan Medical Research Center, The First Affiliated Hospital of Guangzhou University of Chinese Medicine, Guangzhou University of Chinese Medicine, Guangzhou, Guangdong 510000, China; b Department of Orthopaedics & Traumatology, Faculty of Medicine, The Chinese University of Hong Kong, Prince of Wales Hospital, Shatin, Hong Kong SAR 999077, China; c Departments of Diagnostics of Traditional Chinese Medicine, Guangzhou University of Chinese Medicine, Guangzhou, Guangdong 510000, China; d Orthopaedic Research Lab, Affiliated Hospital of Guangdong Medical University, Guangdong Medical University, Zhanjiang, Guangdong 524001, China

Fractures have an extraordinarily negative impact on individuals' quality of life and functional status. Nonunion or disability of fracture is a major health issue with important clinical, social, and economic implications.^1^ Mesenchymal stem cells (MSCs) play an indispensable role in the initiation of the fracture repair process including the formation of a callus which is replaced by new bone. The use of MSCs in the treatment of fractures is very attractive as they can reduce the time of healing and occurrence of nonunion.^2^ However, the effects of MSCs are often hindered by the harsh ischemic micro-environment at the fracture sites, such as low cell survival rate and differentiation *in vivo*.^3^ Histone modifications are one of the most important epigenetic regulations with the ability to control the fate of stem cells. Lysine demethylase 5C (*Kdm5c*) is frequently mutated in patients with X-linked intellectual disabilities, many of whom exhibit physical and behavioral abnormalities, including epilepsy, short stature, *etc*.^4^ In our previous study, we investigated the de-differentiated MSCs with enhanced osteogenic differentiation capacity and found that *Kdm5c* might be involved in regulating the properties of dedifferentiated osteogenic MSCs by PCR array.^5^ However, it is still unclear whether *Kdm5c* plays a role in osteogenesis, bone formation, and fracture repair.

First, we established the *Kdm5c* conventional knockout mice. The MSCs were isolated to evaluate their osteogenic differentiation potential *in vitro*. The data showed that deletion of *Kdm5c* significantly impaired osteogenic differentiation as indicated by decreased mRNA levels of osteogenic markers, including alkaline phosphatase (*Alp*), Runt-related transcription factor 2 (*Runx2*), osteopontin (*Opn*) and osteocalcin (*Ocn*), as well as Alizarin Red S staining **(**Fig. S1). At 5 days and 2 months of age, it was observed that *Kdm5c* knockout (KO) mice had a smaller body size compared to wild-type (WT) littermates (Fig. S2). The whole skeleton of *Kdm5c* KO mice was smaller than that of WT littermates (Fig. 1A). The lengths of the rib, spine (1st thoracic to 1st lumber spine), humerus, ulna, radius, femur, and tibia were shorter in *Kdm5c* KO mice, as compared with that of WT littermates (Fig. 1B–F). The microCT analysis showed *Kdm5c* KO mice exhibited decreased trabecular bone mineral density, bone volume (BV), bone volume fraction (BV/TV), and trabecular number at 8 weeks of age (Fig. 1G, H). Simultaneously, the mineral apposition rate and bone formation rate of both groups were assessed in non-decalcified histological sections of femurs by Calcein-Alizarin Red labeling assay. The endocortical bone formation rate, median mineralizing surface per unit of the bone surface, and endocortical mineral apposition rate were reduced in the *Kdm5c* KO mice compared to WT littermates (Fig. S3A, B), suggesting a decreased endocortical bone formation. The immunohistochemistry (IHC) staining showed that OPG, OCN, and OPN were decreased in the femur's trabecular bone and bone cortex in *Kdm5c* KO mice (Fig. S3C, D).

**Figure 1:**
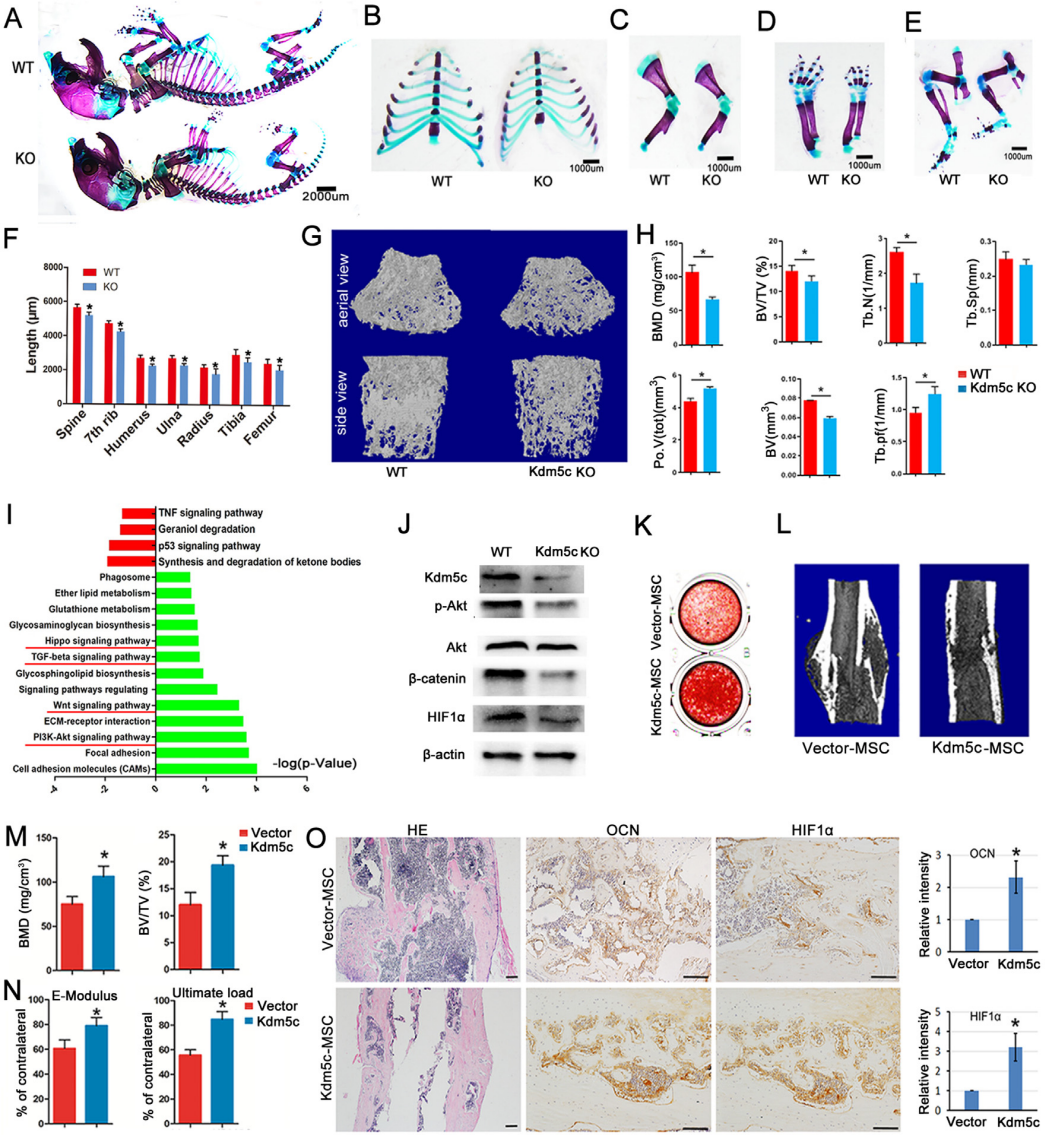
*Kdm5c* deletion impaired bone development by inhibiting PI3K/Akt/HIF1α and Wnt/β-catenin pathways, and its overexpression accelerated fracture healing. **(A–E)** Double staining with Alizarin Red and Alcian Blue of the whole skeleton (A), ribs (B), upper extremities (C, D), and lower extremities (E) of the newborn (Day 1) WT and *Kdm5c* KO mice. Three animals were used in each group. (**F**) The length of the rib, spine (1st thoracic to 1st lumber spine), humerus, ulna, radius, femur, and tibia were calculated. The data are expressed as mean ± standard deviation (SD) (*n* = 3); ∗*P* < 0.05. (**G, H**) Femurs of 2-month-old WT and *Kdm5c* KO mice were analyzed by Micro-CT. The 3D reconstruction of the trabecular bone and quantitative parameters were displayed. The data are expressed as mean ± SD (*n* = 5); ∗*P* < 0.05. (**I**) RNA-seq analysis of gene expression profile of MSCs from WT and *Kdm5c* KO mice. The KEGG analysis showed multiple signaling pathways were inhibited in *Kdm5c* KO MSCs, especially the PI3K/Akt and Wnt signaling pathways. (**J**) Western blot analysis of the relative protein levels of *Kdm5c*, p-Akt, Akt, β-catenin, and HIF1α in MSCs from WT and *Kdm5c* KO mice. (**K**) The *Kdm5c*-MSC and Vector-MSC were treated with an osteogenic induction medium for 14 days, and then the cells were fixed and stained with Alizarin Red S. (**L**) Representative 3D Micro-CT images of the fractured femur of mice treated with *Kdm5c*-MSC and Vector-MSC, respectively. (**M**) The bone mineral density and bone volume fraction (BV/TV) calculated from Micro-CT analysis were displayed. The data are expressed as mean ± SD (*n* = 5); ∗*P* < 0.05. (**N**) Three-point bending mechanical testing at week 4 post-surgery was performed, and the statistical data were displayed (*n* = 5, ∗*P* < 0.05). (**O**) Hematoxylin & eosin (H&E) and immunohistochemistry (IHC) staining with antibodies of OCN and HIF1α. The relative intensity was calculated with Image J. H&E staining: scale bar = 500 μm; IHC staining: scale bar = 100 μm. The data are expressed as mean ± SD (*n* = 5); ∗*P* < 0.05.

Next, we isolated the total RNA of bone marrow MSCs from *Kdm5c* KO and WT mice and performed RNA sequencing (RNAseq) analysis. Among the 724 differentially expressed genes, 112 genes were up-regulated and 612 genes were down-regulated (Fig. S4A). The Kyoto Encyclopedia of Genes and Genomes (KEGG) pathway analysis showed *Kdm5c* KO MSCs were enriched with a variety of signaling pathways closely related to bone development, such as PI3K-Akt, Wnt, Hippo, and Transforming growth factor-β (TGFβ) signaling pathways, *etc* (Fig. 1I). Down-regulation of genes associated with PI3K/Akt and Wnt signaling pathways were shown in Figure S4B. The Western blot analysis also showed the levels of p-Akt, β-catenin, and HIF1α were all decreased in MSCs isolated from *Kdm5c* KO mice (Fig. 1J). In addition, the IHC staining of the tibia revealed significantly reduced p-Akt, β-catenin, and HIF-1α in 2-month-old *Kdm5c* KO mice compared with WT littermates (Fig. S4C).

Then, mouse MSCs were transduced with lentivirus carrying *Kdm5c* or empty vector. Stable cell lines of MSCs (*Kdm5c*-MSC and Vector-MSC) were established. After 5 days of osteogenic induction, the mRNA expression levels of *Alp*, *Opn*, *Bmp2*, *Runx2*, and *Ocn* were significantly increased in *Kdm5c*-MSC (Fig. S5A). The Alizarin Red S staining showed that the mineralization of MSCs was also increased by *Kdm5c* over-expression at 14 days after osteogenic induction (Fig. 1K). The levels of p-Akt, β-catenin, and HIF1α were all increased by *Kdm5c* over-expression (Fig. S5B).

Finally, we established the mouse open femur fracture model, and *Kdm5c*-MSC and Vector-MSC were locally injected into the fracture sites. The Micro-CT result revealed that mice treated with *Kdm5c*-MSC transplantation displayed better fracture healing, significantly higher bone mineral density and bone volume fraction, and better mechanical properties (Fig. 1L–N). Increased levels of OCN and HIF1α in the callus tissue of *Kdm5c*-MSC transplantation mice could be observed (Fig. 1O).

Taken together, our data showed that *Kdm5c* deletion mice displayed decreased bone mass and abnormal bone development, with multiple signaling pathways inhibited, especially the PI3K/Akt/HIF1α and Wnt/β-catenin pathways (summarized in Fig. S6). *Kdm5c*-modified MSCs could be used for accelerating fracture healing, which may have clinical implications for the delayed unions.

## Ethics declaration

All procedures were performed in strict accordance with the guidelines of the Animal Welfare and Ethics Committee of Guangzhou University of Chinese Medicine (No. 20190617017).

## Conflict of interests

The authors declare that they have no conflict of interests with the contents of this manuscript.

## Funding

The work was supported by the National Natural Science Foundation of China (No. 81871778, 81874000, 82272505).

## Data availability

The dataset supporting the conclusions of this work is available in the NCBI Sequence Read Archive (SRA) repository under the submission ID PRJNA890005. The other data that support the findings of this study are available on request from the corresponding authors.
