# Supplementary material for: Histone demethylase Kdm5c regulates osteogenesis and bone formation via PI3K/Akt/HIF1α and Wnt/β-catenin signaling pathways
Source: Genes Dis. 2023 Apr 4;11(1):64–6. doi: 10.1016/j.gendis.2023.02.041 (PMC10425781; doi:10.1016/j.gendis.2023.02.041)
Supplement: Multimedia component 1 [file mmc1.docx]

**Supplementary data**

## Materials and Methods

### Mice

The *Kdm5c* conventional knockout (KO) C57BL/6 mice (Serial Number: KOCMP-23289-*Kdm5c*) were purchased from Cyagen Biosciences (China). As the *Kdm5c* gene is located on the X-chromosome, the hemizygotes display knockout phenotypes, and human individuals affected by the *Kdm5c* gene are predominantly male, so we used male hemizygous mice, namely *Kdm5c* KO and their wild-type littermates (WT). Impaired fertility was observed in *Kdm5c* KO mice.

### Culture of bone marrow MSCs

Generally, the mice of 1-2 months old were sacrificed with an overdose of xylazine and ketamine (Intraperitoneal (IP) injection). According to our previous protocol, bone marrow was flushed out from the bone cavity of the mice ^1^. The bone marrow MSCs were cultured in α-MEM supplemented with 10% FBS (fetal bovine serum), and 2 mM L-glutamine (Invitrogen, USA) at 37℃ with 5% CO_2_. Once the colonies reached 60-80%, they were digested with the usage of trypsin and re-plated for further growth and examination.

### Quantitative polymerase chain reaction (qPCR)

Total RNA was isolated using the RNeasy kit (Qiagen). About 500 ng of total RNA in each sample was reverse transcribed with the Prime Script RT reagent kit (TaKaRa, #RR037A). SYBR Green PCR Master Mix (Vazyme, #Q341) was used for qPCR amplification. Results were calculated using 2^-ΔΔCt^, and relative mRNA expression was calculated. Primers are listed in **Supplementary Table 1**.

### Mineralization assay

The osteogenic induction medium was formulated as previously reported ^2^. MSCs were dealt with or without osteogenic induction medium for 14 days, respectively, and then the cells were fixed and stained with 0.5% Alizarin Red S (pH 4.1). Orange-red staining indicated the location and intensity of calcium deposition.

### Western blot

Proteins were collected, and after measuring the concentration, an equal amount of total protein was loaded onto 10% SDS-PAGE for electrophoresis. After electrophoresis, the proteins were transferred onto a polyvinylidene difluoride (PVDF) membrane (Millipore) using 100 V for approximately 75 minutes. The membrane was blocked and incubated, respectively, with anti-pAkt (Bioss, 1:1000), anti-HIF1α (Santa Cruz, 1:1000), anti-β-catenin (Santa Cruz, 1:1000), anti-KDM5C (Abcam, 1:1000) or anti-β-actin (Santa Cruz, 1:1000) antibodies at 4℃ overnight. After three washes in TBST (5 min each), the PVDF membrane was then incubated with horseradish peroxidase (HRP)-linked secondary antibody. Finally, the proteins were detected with the enhanced chemiluminescence (ECL) blotting reagents (Amersham Biosciences).

### Alcian Blue-Alizarin Red Staining of Mouse Skeleton

Six newborn mice (three mice in each group) were sacrificed with an overdose of xylazine and ketamine. The skeletons were collected and fixed in 95% ethanol. The entire skeletons were soaked for 2 days at room temperature using sufficient Alcian blue solution (0.03% in 80% ethanol and 20% Acetic Acid). The samples were rinsed in ddH_2_O for 2 hours. The samples were counterstained for bone in Alizarin Red solution (0.05% in 0.5 % potassium hydroxide) for 1 day. The samples were rinsed overnight in ddH_2_O and placed in 1% potassium hydroxide for 1 day, and then in a clearing solution (70:30 of 0.5% potassium hydroxide to glycerol) for 1-3 days.

**Calculation of bone formation rate**

Six 4-week-old mice (three mice in each group) were injected intraperitoneally with 20 mg/kg calcein green and 30 mg/kg of Alizarin Red S in a 2% sodium bicarbonate solution. The mice were sacrificed 2 days after the injection of Alizarin Red S with an overdose of xylazine and ketamine (IP injection). The femurs were fixed in 10% buffered PFA, embedded in methyl methacrylate, and sectioned. Images were obtained using a 20× objective fluorescence microscope (LSM 510). Bone formation rate (BFR), mineral apposition rate (MAR), and mineralizing surface per bone surface (MS/BS) were calculated according to the published methods ^3^.

**RNA-seq analysis**

TRIzol™ (Invitrogen, USA) was used to isolate the total RNA of MSCs derived from KDM5C and WT mice. The cDNA library preparation and sequencing were performed by Sangon Biotech (China) according to Illumina's standard protocol. DAVID bioinformatics tool and R package clusterProfiler were used for clustering and functional annotation enrichment analysis. The LIMMA method was used to detect differential expression of genes. The differential genes (absolute log2FoldChange>1, and pvalue<0.05) have been listed in **Supplementary Table 2**.

**Production of lentivirus and infection**

*Kdm5c* was cloned and ligated into a lentiviral vector. Pseudo-lentiviruses were generated by transfection of 293FT cells using lipo3000. At 48 and 72 h after transfection, the culture supernatants were collected to concentrate the lentiviral particles. Then, the lentiviruses were added into culture medium of MSCs, and incubated with 8 mg/ml Polybrene for 24 h in the incubator. To select MSCs stably over-expressing *Kdm5c* or empty vector, puromycin (Invitrogen, USA) was used.

### Micro-CT analysis

The mice were sacrificed with an overdose of xylazine and ketamine (IP injection). Freshly dissected femurs (five per group) were fixed and analyzed using a Skyscan 1176 Micro-CT scanner (Bruke, Belgium) and CT analyzer software. The scanner voltage was 70 kV, the current was 110 mA, and the resolution was 10.8 μm/pixel. The tibia femurs were scanned and parameters such as Bone Mineral Density (BMD), Bone Volume/Tissue Volume (BV/TV), Trabecular Number (Tb.N), Trabecular Separation/Spacing (Tb.sp), total volume of pore space (Po.V (tot)), Bone Volume (BV), and trabecular bone pattern factor (Tb.pf) were measured.

### **Histology**

After euthanasia, mouse bone tissues were harvested and fixed. Bone samples were decalcified with 10% EDTA (PH=7.4) at room temperature for 14 days and shaken continuously before paraffin embedding. Paraffin sections were used for immunohistochemistry and immunofluorescence staining. For immunohistochemistry staining, after appropriate antigen retrieval, slides were incubated with primary antibodies, such as anti-OPN (Abcam, 1:200), anti-OCN (Abcam, 1:200), anti-β-catenin (Santa Cruz, 1:200), anti-HIF1α (Santa Cruz, 1:200), or anti-OPG (Santa Cruz, 1:200) antibodies at 4℃ overnight. After washing in PBS, the slides were incubated with secondary antibodies for 1 h followed by DAB color development. Images were taken with an Olympus BX53 Digital Upright Microscope (Olympus Optical Co., Ltd, Tokyo, Japan) with an Olympus DP 80 camera at 20× (UPLSAPO20×, NA = 0.80, WD = 0.60 mm, Olympus) and 40× objective (UPLSAPO40×, NA = 0.60, WD = 2.7-4.0 mm, Olympus). The image acquisition software cellSens Dimension (Olympus Optical Co., Ltd, Tokyo, Japan) was used for image processing. Five random fields were considered for each immunostaining. The relative intensity calculations were performed using the ImageJ software binarization and thresholding tools.

**Fracture healing model**

Open femoral fracture surgery was performed on 3-month-old mice. Briefly, each mouse was weighed and anesthetized with ketamine (80 mg/kg, IP) and xylazine (12 mg/kg, IP). Then, an incision was made to expose the right femur. A sagittal saw was used to perform a transverse osteotomy of the middle femur. An intramedullary 0.5 mm syringe needle was inserted to fix the fractured femur. Four days later, 50 μL of control (Vector-MSCs) or *Kdm5c* over-expressing MSCs (*Kdm5c* -MSC) were injected into the fracture site. The mice were sacrificed at 4 weeks after surgery. And the femurs were harvested and analyzed by Micro-CT analysis. Then the femurs were fixed with buffered formalin, decalcified, and finally embedded in paraffin for histological analysis.

**Three-Point Bending Mechanical Testing**

Biomechanical assessments were performed with a three-point bending apparatus (H25KS; UK). In general, the femur was placed properly. Failure assessments were carried out on the femur at a constant displacement rate. Testing of the healthy side of the intact femur was also included as control. At the end of the test, the load-displacement curve of the femur was automatically generated by the built-in software. The E-modulus and ultimate load were recorded and analyzed using the built-in software (QMAT Professional Material testing software). The biomechanical properties of femurs were normalized by contralateral intact bone.

### Statistical analysis

SPSS v.11.0 (Chicago, USA) was used for the statistical analysis. The two-tailed Student's t-test was used to determine differences between the groups in this study. P < 0.05 was regarded as a significant difference. A minimum of three independent experimental groups were performed for each analysis.

**
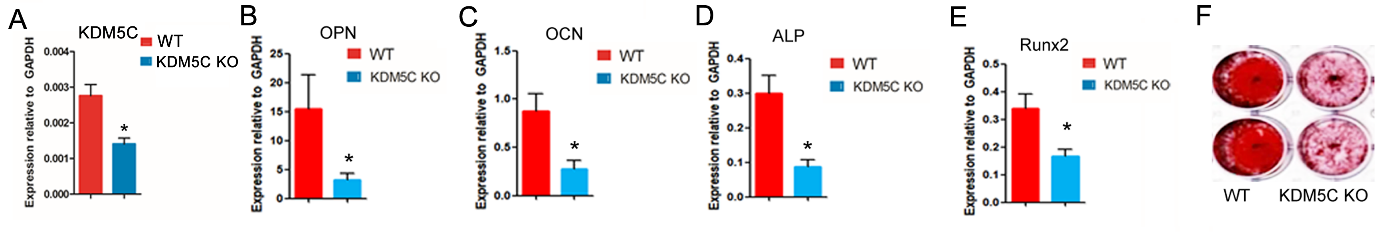
**

**Fig. S1. *Kdm5c* deletion inhibited osteogenic differentiation of MSCs.** **(A-D)** Bone marrow MSCs were isolated and cultured from *Kdm5c* KO and WT mice, the mRNA expression levels of *Kdm5c* and osteogenesis-related genes were evaluated by qPCR. The data are expressed as mean ± SD (n=5), *p < 0.05. **(E)** Representative Alizarin Red S staining for osteogenic differentiation in *Kdm5c* deficient and WT MSCs.

**
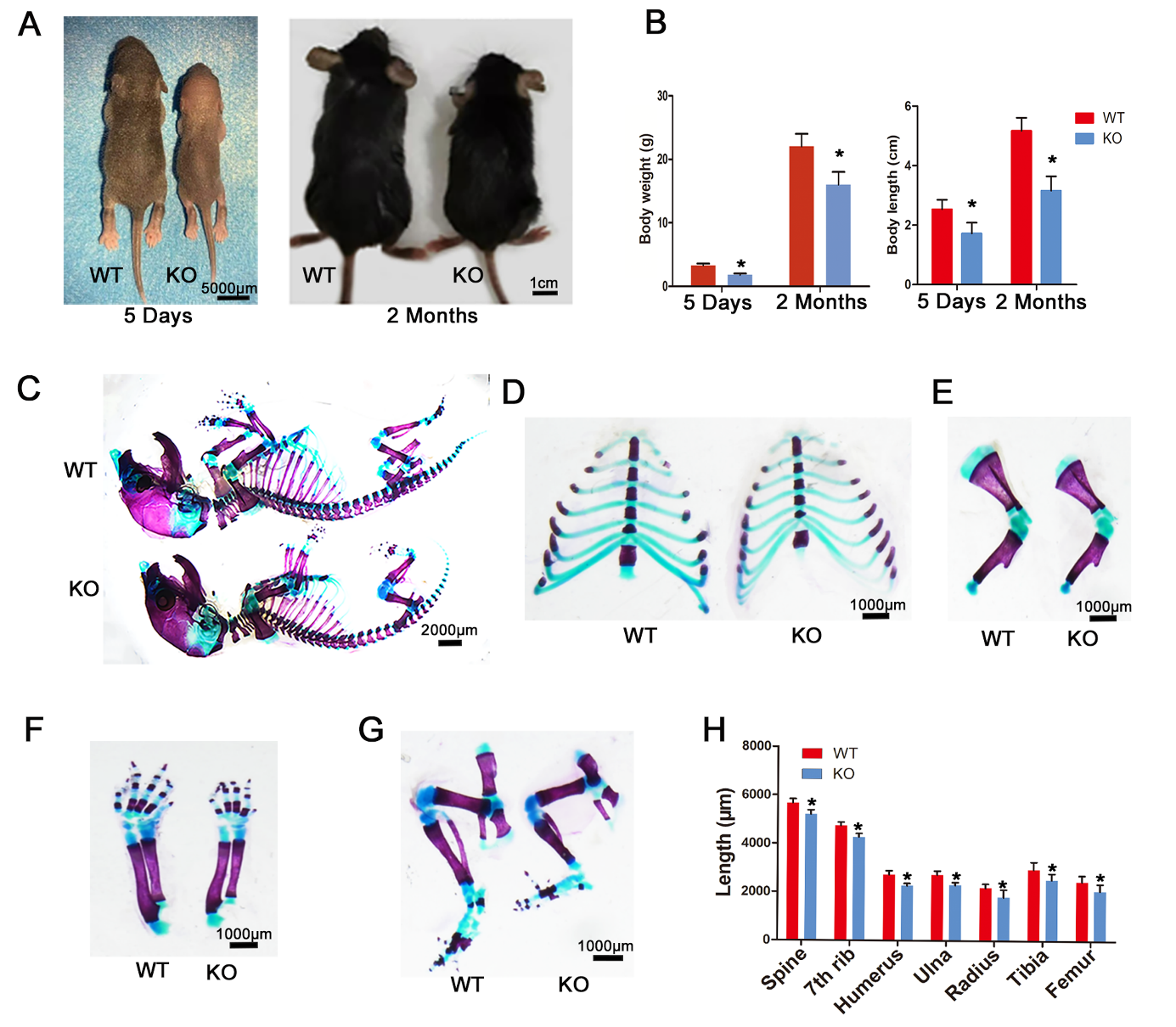
**

**Fig. S2.** ***Kdm5c* deletion impaired bone development.** *Kdm5c* KO mice had a smaller body size at 5 days and 2 months of age. The data are expressed as mean ± SD (n=3), *p < 0.05.

**
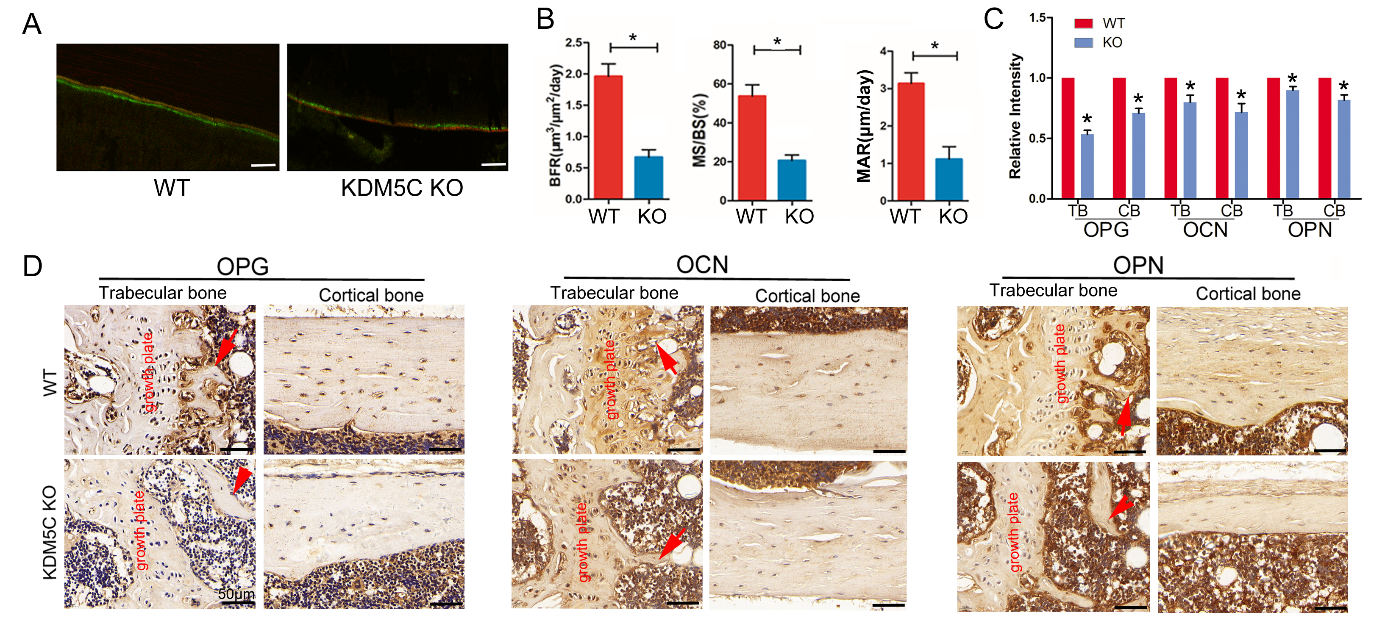
**

**Fig. S3. *Kdm5c* KO mice showed less bone formation. (A&B)** Representative images of Calcein-Alizarin Red double labeling of femora of WT and *Kdm5c* KO mice. BFR, BFR per bone surface (BFR/BS), and mineral apposition rate (MAR) were quantified. The data are expressed as mean ± SD (n=3), *p < 0.05. Scale bar = 25 μm. **(C&D)** Representative immunohistochemistry staining images and semi-quantitative analysis of bone formation markers, including OPG, OCN and OPN in the trabecular bone and cortical bone tissues of 2-month-old WT and KDM5C KO mice. Arrowheads indicated the trabecular bone adjacent to the growth plate. Scale bar = 500 μm. TB, trabecular bone; CB, cortical bone. The data are expressed as mean ± SD (n=5), *p < 0.05.

**
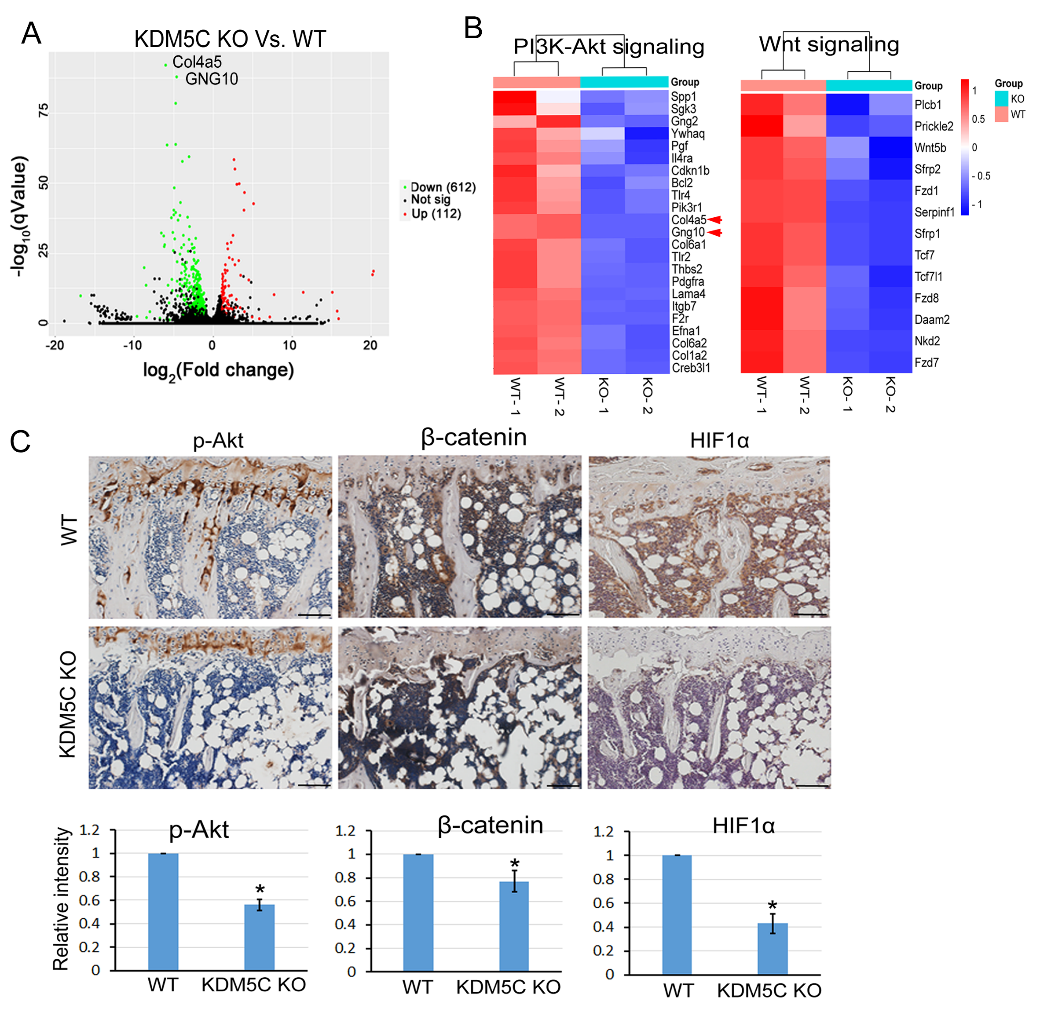
**

**Fig. S4. *Kdm5c* KO inhibited PI3K/Akt/HIF1α and Wnt/β-catenin signaling pathways. (A)** RNA-seq analysis of gene expression profile of MSCs from WT and *Kdm5c* KO mice. Volcano map of the differentially expressed genes (n=2). In total, 724­ genes were differentially expressed in MSCs from WT and *Kdm5c* KO mice. **(B)** Down-regulation of genes associated with PI3K/Akt and Wnt signaling pathways in *Kdm5c* KO MSCs. (**C**) IHC staining analysis to detect p-Akt, β-catenin, and HIF1α in tibia sections of WT and *Kdm5c* KO mice. The relative intensity was calculated with Image J. The data are expressed as mean ± SD (n=5), *p < 0.05. Scale bar = 100 μm.

**
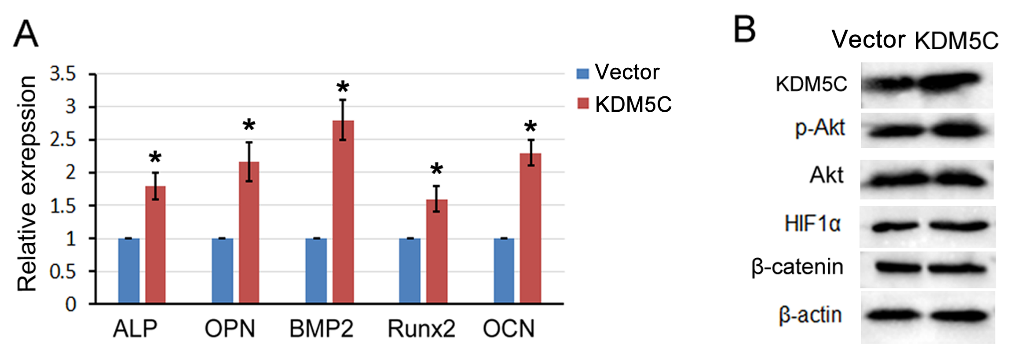
**

**Fig. S5. *Kdm5c* over-expression promoted osteogenesis and activated PI3K/Akt/HIF1α and Wnt/β-catenin pathways. (A)** The *Kdm5c* and vector control stable cell lines (*Kdm5c* -MSC and Vector-MSC) were treated with osteogenic induction medium for 5 days, the mRNA expression levels of ALP, Runx2, BMP2, OPN and OCN were detected by qPCR. The expression level of each gene in the control group was arbitrarily set to 1.0. The fold change of each gene was calculated. The data are expressed as mean ± SD (n=5), *p < 0.05. **(B)** Western blot analysis of the relative protein expression levels of p-Akt, Akt, β-catenin, *Kdm5c* and HIF1α in *Kdm5c* -MSC and Vector-MSC.


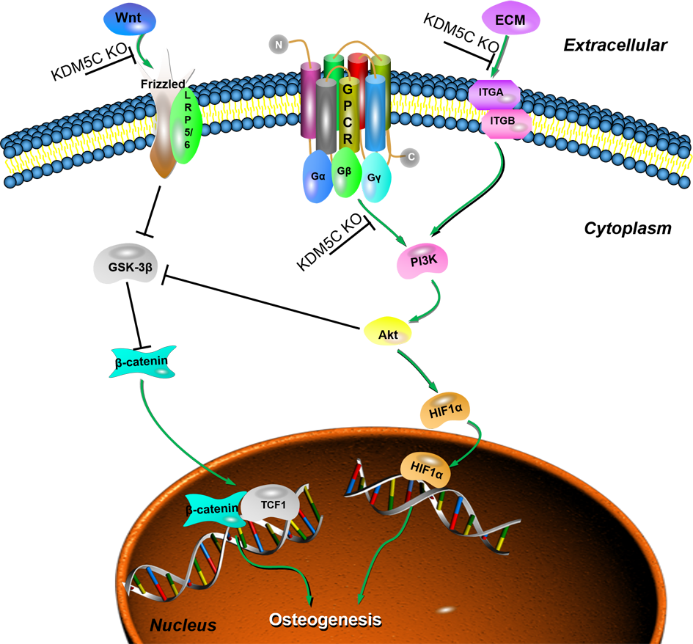


**Fig. S6. Schematic representation of the molecular mechanism of *Kdm5c* regulating bone formation.** The image was generated by the Adobe illustrator 2019 software. In the absence of *Kdm5c*, multiple signaling pathways, especially the PI3K/Akt/HIF1α and Wnt/β-catenin signaling pathways are inhibited, which leads to the suppression of osteogenic differentiation of MSCs, and finally the inhibition of bone formation. An arrow indicates stimulation, whereas a hammerhead indicates inhibition. GPCR, G protein-coupled receptor; ECM, extracellular matrix; PI3K, phosphoinositide 3-kinase; Akt, protein kinase B; HIF1α, hypoxia-inducible factor 1 subunit alpha; Wnt, wingless-type MMTV integration site family.

**Supplementary Table 1.** Sequences of primers for real time qPCR.

| *Gene Name* | *Forward primer sequence (5’ to 3’)* | *Reverse primer sequence (5’ to 3’)* | |
| --- | --- | --- | --- |
| *Alp* | GCAAGGGTGAGGAGGGGTA | CCTCTGAAGGCATTTCATAAGCC |  |
| *Runx2*  *Bmp2* | ATGCTTCATTCGCCTCACAAA  GGGACCCGCTGTCTTCTAGT | GCACTCACTGACTCGGTTGG  TCAACTCAAATTCGCTGAGGAC |  |
| *Ocn* | GGTGGCTTCCGAAGGATTGTC | CCCCCTGATGGGTTGTCAC |  |
| *Opn*  *Gapdh*  *Kdm5c* | AGCAAGAAACTCTTCCAAGCAA  TTGAGGTCAATGAAGGGGTC  GAGGCCCAGACAAGAGTGAAA | GTGAGATTCGTCAGATTCATCCG  TCGTCCCGTAGACAAAATGG TTGGGAATCTTTAAGGATGAGCC |  |

**Supplementary Table 2.** Differentially expressed genes in MSCs from *Kdm5c* KO and WT mice (ordered by pValue).

| Gene id | MeanTPM (KO) | MeanTPM (WT) | log2FoldChange | pValue | qValue | result | GeneName |
| --- | --- | --- | --- | --- | --- | --- | --- |
| ENSMUSG00000031274 | 0.27797 | 18.2791 | -6.03912 | 3.74E-97 | 6.38E-93 | down | Col4a5 |
| ENSMUSG00000038607 | 5.27451 | 132.754 | -4.65358 | 1.45E-92 | 1.23E-88 | down | Gng10 |
| ENSMUSG00000039209 | 5.73168 | 152.122 | -4.73013 | 5.77E-83 | 3.28E-79 | down | Rpl39l |
| ENSMUSG00000013584 | 1.35166 | 36.8531 | -4.76898 | 2.77E-68 | 1.18E-64 | down | Aldh1a2 |
| ENSMUSG00000020875 | 1.01406 | 58.4599 | -5.84923 | 6.53E-68 | 2.23E-64 | down | Hoxb9 |
| ENSMUSG00000000753 | 42.4845 | 353.346 | -3.05607 | 1.04E-63 | 2.94E-60 | down | Serpinf1 |
| ENSMUSG00000024803 | 948.8 | 153.863 | 2.624462 | 1.25E-62 | 3.05E-59 | up | Ankrd1 |
| ENSMUSG00000047250 | 3.38384 | 49.9765 | -3.88452 | 7.26E-62 | 1.55E-58 | down | Ptgs1 |
| ENSMUSG00000016494 | 98.7957 | 14.4535 | 2.773034 | 5.26E-59 | 9.96E-56 | up | Cd34 |
| ENSMUSG00000110981 | 13.2718 | 1.34374 | 3.304034 | 8.20E-54 | 1.40E-50 | up | AC160966.1 |
| ENSMUSG00000063171 | 200.429 | 25.0245 | 3.00168 | 1.26E-53 | 1.95E-50 | up | Rps4l |
| ENSMUSG00000032911 | 0.5849 | 17.5923 | -4.91062 | 2.36E-52 | 3.35E-49 | down | Cspg4 |
| ENSMUSG00000022768 | 12.4444 | 0.80453 | 3.951201 | 1.45E-50 | 1.90E-47 | up | Ccdc116 |
| ENSMUSG00000030691 | 0.75161 | 13.128 | -4.12652 | 6.37E-47 | 7.75E-44 | down | Fchsd2 |
| ENSMUSG00000039410 | 14.0646 | 0.40704 | 5.110742 | 1.99E-46 | 2.25E-43 | up | Prdm16 |
| ENSMUSG00000020467 | 0.6298 | 20.4161 | -5.01867 | 3.27E-44 | 3.48E-41 | down | Efemp1 |
| ENSMUSG00000029121 | 13.5523 | 0.91899 | 3.88235 | 4.67E-44 | 4.68E-41 | up | Crmp1 |
| ENSMUSG00000103585 | 0.32825 | 8.81418 | -4.74696 | 2.25E-43 | 2.13E-40 | down | Pcdhgb4 |
| ENSMUSG00000056427 | 0.20209 | 6.50255 | -5.00794 | 1.61E-42 | 1.44E-39 | down | Slit3 |
| ENSMUSG00000026365 | 1.20257 | 22.5459 | -4.22868 | 2.82E-42 | 2.41E-39 | down | Cfh |
| ENSMUSG00000021567 | 2.82082 | 26.3631 | -3.22433 | 1.34E-41 | 1.09E-38 | down | Nkd2 |
| ENSMUSG00000048763 | 0.33468 | 13.125 | -5.2934 | 2.82E-41 | 2.18E-38 | down | Hoxb3 |
| ENSMUSG00000026579 | 0.23038 | 5.02307 | -4.44646 | 1.58E-40 | 1.17E-37 | down | F5 |
| ENSMUSG00000025332 | 4.49438 | 36.0271 | -3.00289 | 4.35E-38 | 3.09E-35 | down | Kdm5c |
| ENSMUSG00000000690 | 0.5582 | 19.1182 | -5.09802 | 5.11E-36 | 3.48E-33 | down | Hoxb6 |
| ENSMUSG00000036782 | 0.12627 | 11.8526 | -6.55258 | 1.06E-35 | 6.97E-33 | down | Klhl13 |
| ENSMUSG00000097289 | 29.6819 | 5.18152 | 2.518133 | 4.83E-35 | 3.05E-32 | up | 2010300F17Rik |
| ENSMUSG00000027199 | 1.94847 | 26.7968 | -3.78165 | 5.91E-35 | 3.60E-32 | down | Gatm |
| ENSMUSG00000038721 | 0.30902 | 23.3853 | -6.24176 | 9.45E-35 | 5.55E-32 | down | Hoxb7 |
| ENSMUSG00000042793 | 16.5002 | 77.9628 | -2.2403 | 6.51E-34 | 3.69E-31 | down | Lgr6 |
| ENSMUSG00000015568 | 0.22362 | 6.49712 | -4.86069 | 1.16E-33 | 6.40E-31 | down | Lpl |
| ENSMUSG00000039062 | 4.16629 | 34.6425 | -3.05571 | 2.41E-33 | 1.28E-30 | down | Anpep |
| ENSMUSG00000085183 | 161.031 | 33.2247 | 2.277008 | 2.72E-32 | 1.41E-29 | up | Gm12603 |
| ENSMUSG00000045954 | 153.946 | 45.0495 | 1.772845 | 8.35E-32 | 4.18E-29 | up | Sdpr |
| ENSMUSG00000031548 | 3.05488 | 20.1327 | -2.72035 | 1.19E-31 | 5.81E-29 | down | Sfrp1 |
| ENSMUSG00000019846 | 0.52655 | 7.08444 | -3.75001 | 1.88E-31 | 8.88E-29 | down | Lama4 |
| ENSMUSG00000057722 | 0.07191 | 5.06805 | -6.13914 | 2.07E-31 | 9.51E-29 | down | Lepr |
| ENSMUSG00000030220 | 0.39921 | 29.4763 | -6.20626 | 1.01E-30 | 4.52E-28 | down | Arhgdib |
| ENSMUSG00000029108 | 0.8213 | 13.2296 | -4.00972 | 1.93E-30 | 8.42E-28 | down | Pcdh7 |
| ENSMUSG00000052957 | 16.1901 | 85.9158 | -2.40781 | 2.54E-30 | 1.08E-27 | down | Gas1 |
| ENSMUSG00000030218 | 48.3942 | 292.099 | -2.59355 | 3.71E-30 | 1.54E-27 | down | Mgp |
| ENSMUSG00000018339 | 31.685 | 195.715 | -2.62689 | 4.13E-30 | 1.68E-27 | down | Gpx3 |
| ENSMUSG00000032085 | 1775.4 | 497.599 | 1.835084 | 5.18E-30 | 2.05E-27 | up | Tagln |
| ENSMUSG00000010476 | 0.23092 | 7.55302 | -5.03158 | 2.23E-29 | 8.63E-27 | down | Ebf3 |
| ENSMUSG00000050578 | 2.02142 | 18.3659 | -3.18359 | 5.39E-29 | 1.99E-26 | down | Mmp13 |
| ENSMUSG00000032024 | 5.66192 | 30.1221 | -2.41146 | 1.21E-28 | 4.30E-26 | down | Clmp |
| ENSMUSG00000004151 | 0.48481 | 9.11019 | -4.232 | 6.09E-28 | 2.12E-25 | down | Etv1 |
| ENSMUSG00000062345 | 79.8901 | 19.9059 | 2.004817 | 1.23E-26 | 4.17E-24 | up | Serpinb2 |
| ENSMUSG00000027253 | 1.92571 | 10.4159 | -2.43533 | 3.67E-26 | 1.21E-23 | down | Lrp4 |
| ENSMUSG00000063767 | 29.798 | 5.53247 | 2.429217 | 3.92E-26 | 1.26E-23 | up | S100a7a |
| ENSMUSG00000020644 | 34.5674 | 129.816 | -1.90898 | 1.15E-25 | 3.61E-23 | down | Id2 |
| ENSMUSG00000023092 | 110.546 | 11.9185 | 3.213375 | 1.21E-25 | 3.74E-23 | up | Fhl1 |
| ENSMUSG00000021806 | 3.9326 | 20.89 | -2.40926 | 1.31E-25 | 3.97E-23 | down | Nid2 |
| ENSMUSG00000052981 | 0.36795 | 8.94917 | -4.60417 | 4.03E-25 | 1.21E-22 | down | Ube2ql1 |
| ENSMUSG00000074743 | 19.0816 | 80.8039 | -2.08224 | 4.85E-25 | 1.40E-22 | down | Thbd |
| ENSMUSG00000047878 | 1.29558 | 13.4482 | -3.37575 | 6.15E-25 | 1.74E-22 | down | A4galt |
| ENSMUSG00000026620 | 5.05182 | 23.6601 | -2.22758 | 1.27E-24 | 3.53E-22 | down | Mark1 |
| ENSMUSG00000061048 | 0.4203 | 6.39919 | -3.92841 | 2.89E-24 | 7.93E-22 | down | Cdh3 |
| ENSMUSG00000090877 | 72.4048 | 25.1185 | 1.527336 | 3.52E-24 | 9.51E-22 | up | Hspa1b |
| ENSMUSG00000054863 | 0.80196 | 10.0391 | -3.64595 | 3.94E-24 | 1.05E-21 | down | Fam19a5 |
| ENSMUSG00000071042 | 5.24614 | 0.78614 | 2.738392 | 5.11E-24 | 1.34E-21 | up | Rasgrp3 |
| ENSMUSG00000074643 | 7.5167 | 42.8794 | -2.51211 | 2.12E-23 | 5.46E-21 | down | Cpne1 |
| ENSMUSG00000026814 | 2.83461 | 18.4606 | -2.70323 | 2.68E-23 | 6.82E-21 | down | Eng |
| ENSMUSG00000050069 | 0.56083 | 6.63411 | -3.56428 | 3.20E-23 | 8.01E-21 | down | Grem2 |
| ENSMUSG00000075588 | 0.02599 | 10.9847 | -8.72327 | 5.16E-23 | 1.27E-20 | down | Hoxb2 |
| ENSMUSG00000022425 | 2.85684 | 17.2416 | -2.59341 | 1.29E-22 | 3.14E-20 | down | Enpp2 |
| ENSMUSG00000038456 | 0.88601 | 9.40725 | -3.40837 | 1.32E-22 | 3.16E-20 | down | Dennd2a |
| ENSMUSG00000053062 | 2.71741 | 14.4932 | -2.41508 | 2.03E-22 | 4.79E-20 | down | Jam2 |
| ENSMUSG00000035493 | 2.15303 | 14.8299 | -2.78407 | 2.58E-22 | 6.01E-20 | down | Tgfbi |
| ENSMUSG00000037411 | 1069 | 382.786 | 1.481657 | 3.35E-22 | 7.70E-20 | up | Serpine1 |
| ENSMUSG00000059430 | 298.744 | 86.8526 | 1.782268 | 5.69E-22 | 1.29E-19 | up | Actg2 |
| ENSMUSG00000039114 | 59.7079 | 260.576 | -2.12571 | 7.53E-22 | 1.69E-19 | down | Nrn1 |
| ENSMUSG00000028766 | 1.27473 | 15.2069 | -3.57646 | 1.02E-21 | 2.26E-19 | down | Alpl |
| ENSMUSG00000031125 | 131.583 | 0.0001 | 20.32754 | 1.21E-21 | 2.64E-19 | up | 3830403N18Rik |
| ENSMUSG00000023886 | 20.9652 | 79.9772 | -1.93159 | 1.62E-21 | 3.49E-19 | down | Smoc2 |
| ENSMUSG00000000958 | 0.55476 | 8.87971 | -4.00059 | 4.16E-21 | 8.86E-19 | down | Slc7a7 |
| ENSMUSG00000074578 | 213.532 | 74.7133 | 1.515017 | 4.72E-21 | 9.91E-19 | up | Zfas1 |
| ENSMUSG00000024084 | 17.252 | 69.2702 | -2.00547 | 9.00E-21 | 1.87E-18 | down | Qpct |
| ENSMUSG00000028364 | 293.327 | 131.538 | 1.15703 | 1.30E-20 | 2.67E-18 | up | Tnc |
| ENSMUSG00000045410 | 3.34182 | 18.9322 | -2.50214 | 1.40E-20 | 2.84E-18 | down | Akr1e1 |
| ENSMUSG00000096768 | 120.939 | 0.0001 | 20.20585 | 1.67E-20 | 3.36E-18 | up | Erdr1 |
| ENSMUSG00000027230 | 20.4811 | 77.3266 | -1.91667 | 1.83E-20 | 3.62E-18 | down | Creb3l1 |
| ENSMUSG00000000627 | 0.55461 | 6.08873 | -3.45659 | 1.90E-20 | 3.73E-18 | down | Sema4f |
| ENSMUSG00000031309 | 13.7002 | 45.6253 | -1.73563 | 2.16E-20 | 4.18E-18 | down | Rps6ka3 |
| ENSMUSG00000026494 | 2.29564 | 9.6138 | -2.06621 | 2.60E-20 | 4.98E-18 | down | Kif26b |
| ENSMUSG00000021200 | 5.23121 | 0.46686 | 3.486071 | 3.46E-20 | 6.54E-18 | up | Asb2 |
| ENSMUSG00000029231 | 2.24227 | 9.85033 | -2.13522 | 4.63E-20 | 8.66E-18 | down | Pdgfra |
| ENSMUSG00000002985 | 3.36909 | 28.5913 | -3.08515 | 5.17E-20 | 9.56E-18 | down | Apoe |
| ENSMUSG00000030468 | 69.7505 | 29.7832 | 1.227705 | 1.06E-19 | 1.94E-17 | up | Siglecg |
| ENSMUSG00000031375 | 482.365 | 1600.43 | -1.73026 | 1.55E-19 | 2.77E-17 | down | Bgn |
| ENSMUSG00000020121 | 1.22507 | 7.03297 | -2.52127 | 2.04E-19 | 3.63E-17 | down | Srgap1 |
| ENSMUSG00000045362 | 0.46137 | 5.64207 | -3.61224 | 4.10E-19 | 7.20E-17 | down | Tnfrsf26 |
| ENSMUSG00000019066 | 3.07062 | 14.2033 | -2.20963 | 5.11E-19 | 8.87E-17 | down | Rab3d |
| ENSMUSG00000019997 | 1608.13 | 723.127 | 1.153063 | 5.79E-19 | 9.96E-17 | up | Ctgf |
| ENSMUSG00000027204 | 13.0164 | 40.3087 | -1.63076 | 6.74E-19 | 1.12E-16 | down | Fbn1 |
| ENSMUSG00000036564 | 7.61855 | 34.6931 | -2.18706 | 9.41E-19 | 1.56E-16 | down | Ndrg4 |
| ENSMUSG00000063506 | 21.7761 | 81.1882 | -1.89852 | 9.66E-19 | 1.58E-16 | down | Arhgap22 |
| ENSMUSG00000026580 | 0.15634 | 5.84127 | -5.22355 | 1.09E-18 | 1.76E-16 | down | Selp |
| ENSMUSG00000067276 | 6.2532 | 24.938 | -1.99568 | 1.17E-18 | 1.87E-16 | down | Capn6 |
| ENSMUSG00000031980 | 0.52661 | 8.38615 | -3.99319 | 1.37E-18 | 2.16E-16 | down | Agt |
| ENSMUSG00000108584 | 16.78 | 0.73134 | 4.52006 | 2.00E-18 | 3.13E-16 | up | Gm45216 |
| ENSMUSG00000020598 | 0.59526 | 5.94759 | -3.32071 | 2.06E-18 | 3.19E-16 | down | Nrcam |
| ENSMUSG00000001504 | 23.1457 | 7.89767 | 1.551247 | 2.81E-18 | 4.31E-16 | up | Irx2 |
| ENSMUSG00000036545 | 6.38538 | 22.6322 | -1.82553 | 2.93E-18 | 4.46E-16 | down | Adamts2 |
| ENSMUSG00000054196 | 0.43176 | 10.4474 | -4.59677 | 4.85E-18 | 7.31E-16 | down | Cthrc1 |
| ENSMUSG00000036523 | 1.40983 | 7.41037 | -2.39402 | 6.97E-18 | 1.04E-15 | down | Greb1 |
| ENSMUSG00000019970 | 158.706 | 68.0038 | 1.22267 | 8.60E-18 | 1.27E-15 | up | Sgk1 |
| ENSMUSG00000031841 | 5.21845 | 21.1141 | -2.01651 | 2.46E-17 | 3.58E-15 | down | Cdh13 |
| ENSMUSG00000091387 | 0.50949 | 5.19596 | -3.35027 | 2.56E-17 | 3.69E-15 | down | Gcnt4 |
| ENSMUSG00000019894 | 0.93605 | 7.42281 | -2.98731 | 3.12E-17 | 4.47E-15 | down | Slc6a15 |
| ENSMUSG00000029710 | 6.50948 | 26.2125 | -2.00964 | 4.24E-17 | 6.02E-15 | down | Ephb4 |
| ENSMUSG00000038692 | 0.57295 | 7.03814 | -3.61872 | 4.45E-17 | 6.26E-15 | down | Hoxb4 |
| ENSMUSG00000042834 | 0.65606 | 7.76658 | -3.56539 | 6.72E-17 | 9.38E-15 | down | Nrep |
| ENSMUSG00000035131 | 0.27256 | 5.04072 | -4.209 | 8.70E-17 | 1.20E-14 | down | Brinp3 |
| ENSMUSG00000022674 | 40.5449 | 18.0653 | 1.166299 | 1.01E-16 | 1.39E-14 | up | Ube2v2 |
| ENSMUSG00000024371 | 4.37093 | 21.2197 | -2.27939 | 1.47E-16 | 2.00E-14 | down | C2 |
| ENSMUSG00000005583 | 0.13389 | 7.01742 | -5.7118 | 1.59E-16 | 2.15E-14 | down | Mef2c |
| ENSMUSG00000064373 | 12.3 | 49.6956 | -2.01446 | 1.60E-16 | 2.15E-14 | down | Selenop |
| ENSMUSG00000073125 | 0.02631 | 9.59927 | -8.51136 | 2.15E-16 | 2.86E-14 | down | Xlr3b |
| ENSMUSG00000040612 | 3.62616 | 12.7546 | -1.8145 | 2.36E-16 | 3.11E-14 | down | Ildr2 |
| ENSMUSG00000049422 | 7.67771 | 37.6268 | -2.29301 | 2.63E-16 | 3.45E-14 | down | Chchd10 |
| ENSMUSG00000097287 | 0.40476 | 6.02874 | -3.89673 | 2.68E-16 | 3.49E-14 | down | D130017N08Rik |
| ENSMUSG00000029377 | 17.4402 | 4.38272 | 1.992519 | 3.15E-16 | 4.06E-14 | up | Ereg |
| ENSMUSG00000047139 | 36.1884 | 114.326 | -1.65956 | 4.04E-16 | 5.17E-14 | down | Cd24a |
| ENSMUSG00000033213 | 2.88175 | 29.1756 | -3.33975 | 5.07E-16 | 6.39E-14 | down | AA467197 |
| ENSMUSG00000029838 | 2.83391 | 19.5743 | -2.7881 | 5.11E-16 | 6.39E-14 | down | Ptn |
| ENSMUSG00000078923 | 101.826 | 35.3708 | 1.525469 | 5.09E-16 | 6.39E-14 | up | Ube2v1 |
| ENSMUSG00000001768 | 8.04333 | 26.6792 | -1.72985 | 5.83E-16 | 7.20E-14 | down | Rin2 |
| ENSMUSG00000108414 | 303.014 | 126.835 | 1.256427 | 6.70E-16 | 8.21E-14 | up | Snhg1 |
| ENSMUSG00000019124 | 7.29604 | 53.3389 | -2.87 | 8.36E-16 | 1.02E-13 | down | Scrn1 |
| ENSMUSG00000031673 | 24.2757 | 76.78 | -1.66122 | 9.45E-16 | 1.13E-13 | down | Cdh11 |
| ENSMUSG00000025978 | 3.19129 | 11.5156 | -1.85138 | 1.03E-15 | 1.23E-13 | down | Rftn2 |
| ENSMUSG00000020601 | 0.94674 | 7.56519 | -2.99833 | 1.40E-15 | 1.66E-13 | down | Trib2 |
| ENSMUSG00000087590 | 97.7372 | 34.4872 | 1.502845 | 1.62E-15 | 1.91E-13 | up | Epb41l4aos |
| ENSMUSG00000020205 | 180.252 | 81.7514 | 1.140699 | 2.14E-15 | 2.50E-13 | up | Phlda1 |
| ENSMUSG00000039005 | 3.02168 | 14.3459 | -2.24721 | 3.23E-15 | 3.74E-13 | down | Tlr4 |
| ENSMUSG00000048387 | 22.088 | 71.6511 | -1.69773 | 3.48E-15 | 4.00E-13 | down | Osr1 |
| ENSMUSG00000054675 | 2.34168 | 14.2187 | -2.60218 | 3.87E-15 | 4.39E-13 | down | Tmem119 |
| ENSMUSG00000017446 | 3.13395 | 15.1154 | -2.26996 | 6.37E-15 | 7.13E-13 | down | C1qtnf1 |
| ENSMUSG00000023391 | 10.8328 | 39.0887 | -1.85134 | 6.93E-15 | 7.71E-13 | down | Dlx2 |
| ENSMUSG00000034612 | 19.2529 | 59.7042 | -1.63275 | 8.19E-15 | 9.06E-13 | down | Chst11 |
| ENSMUSG00000011256 | 5.01668 | 17.727 | -1.82114 | 1.03E-14 | 1.14E-12 | down | Adam19 |
| ENSMUSG00000038859 | 1.27734 | 7.69843 | -2.59142 | 1.08E-14 | 1.18E-12 | down | Baiap2l1 |
| ENSMUSG00000032487 | 96.8569 | 38.7165 | 1.322907 | 1.16E-14 | 1.26E-12 | up | Ptgs2 |
| ENSMUSG00000036377 | 2.30182 | 11.0841 | -2.26765 | 1.19E-14 | 1.29E-12 | down | C530008M17Rik |
| ENSMUSG00000031355 | 0.67206 | 6.07914 | -3.17721 | 1.31E-14 | 1.40E-12 | down | Arhgap6 |
| ENSMUSG00000021186 | 15.3333 | 57.2087 | -1.89956 | 1.60E-14 | 1.70E-12 | down | Fbln5 |
| ENSMUSG00000041757 | 1.65922 | 8.75136 | -2.399 | 1.93E-14 | 2.02E-12 | down | Plekha6 |
| ENSMUSG00000032076 | 0.15463 | 5.70762 | -5.20602 | 1.98E-14 | 2.04E-12 | down | Cadm1 |
| ENSMUSG00000048376 | 31.0519 | 90.1492 | -1.53764 | 1.97E-14 | 2.04E-12 | down | F2r |
| ENSMUSG00000045092 | 13.2264 | 44.8358 | -1.76123 | 2.67E-14 | 2.72E-12 | down | S1pr1 |
| ENSMUSG00000000031 | 0.37514 | 6.05457 | -4.01252 | 3.11E-14 | 3.13E-12 | down | H19 |
| ENSMUSG00000002688 | 1.30213 | 7.0767 | -2.44221 | 4.10E-14 | 4.08E-12 | down | Prkd1 |
| ENSMUSG00000021367 | 53.4794 | 25.1674 | 1.087429 | 4.09E-14 | 4.08E-12 | up | Edn1 |
| ENSMUSG00000032420 | 4.66565 | 16.8212 | -1.85013 | 4.90E-14 | 4.85E-12 | down | Nt5e |
| ENSMUSG00000029500 | 82.2609 | 37.2026 | 1.144805 | 5.51E-14 | 5.42E-12 | up | Pgam5 |
| ENSMUSG00000038175 | 5.25252 | 18.9557 | -1.85155 | 7.36E-14 | 7.16E-12 | down | Mylip |
| ENSMUSG00000004951 | 318.803 | 136.3 | 1.225883 | 9.13E-14 | 8.84E-12 | up | Hspb1 |
| ENSMUSG00000075592 | 4.11758 | 14.0461 | -1.77031 | 9.35E-14 | 8.99E-12 | down | Nynrin |
| ENSMUSG00000095562 | 77.9897 | 0.02896 | 11.39513 | 9.47E-14 | 9.06E-12 | up | Gm21887 |
| ENSMUSG00000098387 | 18.1384 | 0.00052 | 15.09296 | 1.07E-13 | 1.01E-11 | up | Pet117 |
| ENSMUSG00000021196 | 20.6633 | 46.8694 | -1.18157 | 1.13E-13 | 1.07E-11 | down | Pfkp |
| ENSMUSG00000040111 | 2.15486 | 9.19459 | -2.09319 | 1.41E-13 | 1.32E-11 | down | Gramd1b |
| ENSMUSG00000025161 | 131.127 | 55.073 | 1.251549 | 1.61E-13 | 1.49E-11 | up | Slc16a3 |
| ENSMUSG00000035274 | 15.7811 | 6.1997 | 1.347923 | 1.64E-13 | 1.51E-11 | up | Tpbg |
| ENSMUSG00000026833 | 2.92791 | 11.6633 | -1.99402 | 2.43E-13 | 2.24E-11 | down | Olfm1 |
| ENSMUSG00000027966 | 3.44225 | 12.43 | -1.85241 | 2.54E-13 | 2.32E-11 | down | Col11a1 |
| ENSMUSG00000047045 | 6.12515 | 14.4402 | -1.23727 | 3.00E-13 | 2.72E-11 | down | Tmem164 |
| ENSMUSG00000034488 | 0.93341 | 5.51869 | -2.56375 | 3.29E-13 | 2.94E-11 | down | Edil3 |
| ENSMUSG00000044674 | 10.0494 | 31.1731 | -1.63319 | 3.30E-13 | 2.94E-11 | down | Fzd1 |
| ENSMUSG00000068747 | 7.85255 | 23.9113 | -1.60646 | 3.98E-13 | 3.53E-11 | down | Sort1 |
| ENSMUSG00000025026 | 6.92045 | 23.1799 | -1.74394 | 4.62E-13 | 4.08E-11 | down | Add3 |
| ENSMUSG00000031239 | 8.60415 | 31.7268 | -1.8826 | 5.47E-13 | 4.78E-11 | down | Itm2a |
| ENSMUSG00000021381 | 8.10272 | 0.03848 | 7.718134 | 5.45E-13 | 4.78E-11 | up | Barx1 |
| ENSMUSG00000039116 | 6.67544 | 20.2194 | -1.59881 | 5.51E-13 | 4.79E-11 | down | Adgrg6 |
| ENSMUSG00000001349 | 52.4722 | 21.3667 | 1.296188 | 5.77E-13 | 4.99E-11 | up | Cnn1 |
| ENSMUSG00000005397 | 80.9187 | 208.305 | -1.36415 | 5.88E-13 | 5.06E-11 | down | Nid1 |
| ENSMUSG00000007888 | 43.1241 | 18.2843 | 1.237887 | 6.35E-13 | 5.44E-11 | up | Crlf1 |
| ENSMUSG00000015053 | 14.0925 | 49.9188 | -1.82465 | 6.94E-13 | 5.90E-11 | down | Gata2 |
| ENSMUSG00000020432 | 34.1034 | 98.6991 | -1.53312 | 6.97E-13 | 5.90E-11 | down | Tcn2 |
| ENSMUSG00000006403 | 3.19005 | 11.4095 | -1.83859 | 8.33E-13 | 6.98E-11 | down | Adamts4 |
| ENSMUSG00000021728 | 16.3127 | 51.4275 | -1.65654 | 9.92E-13 | 8.28E-11 | down | Emb |
| ENSMUSG00000021214 | 1.00334 | 13.0443 | -3.70053 | 1.04E-12 | 8.61E-11 | down | Akr1c18 |
| ENSMUSG00000079852 | 67.1283 | 31.4066 | 1.095855 | 1.04E-12 | 8.61E-11 | up | Klra4 |
| ENSMUSG00000052760 | 12.0664 | 2.36076 | 2.353669 | 1.27E-12 | 1.03E-10 | up | A630001G21Rik |
| ENSMUSG00000050732 | 51.2891 | 183.734 | -1.8409 | 1.46E-12 | 1.18E-10 | down | Vamp8 |
| ENSMUSG00000041073 | 1.11442 | 5.78165 | -2.37519 | 1.65E-12 | 1.32E-10 | down | Nacad |
| ENSMUSG00000034777 | 0.48829 | 11.1931 | -4.51872 | 1.70E-12 | 1.35E-10 | down | Vax2 |
| ENSMUSG00000022231 | 3.52332 | 11.4564 | -1.70114 | 1.86E-12 | 1.47E-10 | down | Sema5a |
| ENSMUSG00000078706 | 0.0001 | 11.3987 | -16.7985 | 2.00E-12 | 1.57E-10 | down | Gm53 |
| ENSMUSG00000026414 | 19.7049 | 4.5998 | 2.098909 | 2.09E-12 | 1.64E-10 | up | Tnnt2 |
| ENSMUSG00000028184 | 2.54333 | 12.3513 | -2.27987 | 2.31E-12 | 1.80E-10 | down | Adgrl2 |
| ENSMUSG00000078452 | 8.73979 | 35.7555 | -2.0325 | 3.20E-12 | 2.45E-10 | down | Raet1d |
| ENSMUSG00000058070 | 15.1244 | 43.295 | -1.51733 | 3.21E-12 | 2.45E-10 | down | Eml1 |
| ENSMUSG00000029641 | 4.18868 | 21.1648 | -2.3371 | 3.59E-12 | 2.70E-10 | down | Rasl11a |
| ENSMUSG00000044134 | 3.40826 | 10.4209 | -1.61237 | 3.63E-12 | 2.72E-10 | down | Fam109a |
| ENSMUSG00000001435 | 3.91215 | 11.0705 | -1.50068 | 4.13E-12 | 3.06E-10 | down | Col18a1 |
| ENSMUSG00000018102 | 13.2749 | 56.3722 | -2.08629 | 8.07E-12 | 5.92E-10 | down | Hist1h2bc |
| ENSMUSG00000030671 | 4.43745 | 14.0964 | -1.66753 | 8.92E-12 | 6.52E-10 | down | Pde3b |
| ENSMUSG00000001281 | 7.59153 | 23.7232 | -1.64384 | 9.71E-12 | 7.07E-10 | down | Itgb7 |
| ENSMUSG00000051920 | 2.20704 | 9.02177 | -2.0313 | 1.08E-11 | 7.78E-10 | down | Rspo2 |
| ENSMUSG00000001119 | 38.0472 | 100.199 | -1.397 | 1.23E-11 | 8.83E-10 | down | Col6a1 |
| ENSMUSG00000111521 | 31.69 | 11.046 | 1.520509 | 1.36E-11 | 9.72E-10 | up | AC122273.2 |
| ENSMUSG00000041695 | 1.12941 | 5.14762 | -2.18833 | 1.51E-11 | 1.06E-09 | down | Kcnj2 |
| ENSMUSG00000086841 | 537.069 | 247.403 | 1.118245 | 1.78E-11 | 1.25E-09 | up | 2410006H16Rik |
| ENSMUSG00000007908 | 16.4882 | 6.35221 | 1.376105 | 1.79E-11 | 1.25E-09 | up | Hmgcll1 |
| ENSMUSG00000023046 | 1.53512 | 16.1022 | -3.39084 | 1.87E-11 | 1.30E-09 | down | Igfbp6 |
| ENSMUSG00000021765 | 2.77712 | 11.896 | -2.09882 | 2.13E-11 | 1.48E-09 | down | Fst |
| ENSMUSG00000027603 | 8.02644 | 28.69 | -1.83771 | 2.26E-11 | 1.56E-09 | down | Ggt7 |
| ENSMUSG00000013415 | 2.30029 | 7.1999 | -1.64616 | 2.40E-11 | 1.65E-09 | down | Igf2bp1 |
| ENSMUSG00000037405 | 0.65899 | 5.00608 | -2.92535 | 2.50E-11 | 1.71E-09 | down | Icam1 |
| ENSMUSG00000029126 | 37.8937 | 151.436 | -1.99868 | 2.63E-11 | 1.78E-09 | down | Nsg1 |
| ENSMUSG00000026586 | 37.2568 | 119.283 | -1.67881 | 2.62E-11 | 1.78E-09 | down | Prrx1 |
| ENSMUSG00000037846 | 3.16102 | 12.4928 | -1.98264 | 2.73E-11 | 1.85E-09 | down | Rtkn2 |
| ENSMUSG00000029661 | 244.201 | 591.342 | -1.27592 | 3.11E-11 | 2.09E-09 | down | Col1a2 |
| ENSMUSG00000036743 | 0.5454 | 7.10643 | -3.70373 | 3.30E-11 | 2.20E-09 | down | Psma8 |
| ENSMUSG00000086290 | 103.824 | 40.7002 | 1.351032 | 3.40E-11 | 2.25E-09 | up | Snhg12 |
| ENSMUSG00000033060 | 4.35695 | 13.5092 | -1.63255 | 3.44E-11 | 2.27E-09 | down | Lmo7 |
| ENSMUSG00000033420 | 46.1983 | 120.279 | -1.38047 | 4.14E-11 | 2.71E-09 | down | Antxr1 |
| ENSMUSG00000063450 | 7.44615 | 25.6538 | -1.7846 | 4.35E-11 | 2.84E-09 | down | Syne2 |
| ENSMUSG00000028517 | 3.55972 | 14.5282 | -2.02902 | 4.54E-11 | 2.93E-09 | down | Plpp3 |
| ENSMUSG00000101581 | 12.0406 | 1.56298 | 2.945531 | 5.04E-11 | 3.21E-09 | up | C430002N11Rik |
| ENSMUSG00000038991 | 147.857 | 380.823 | -1.36492 | 5.06E-11 | 3.22E-09 | down | Txndc5 |
| ENSMUSG00000055172 | 16.9134 | 46.706 | -1.46545 | 5.82E-11 | 3.67E-09 | down | C1ra |
| ENSMUSG00000031734 | 8.3006 | 26.9674 | -1.69993 | 7.02E-11 | 4.41E-09 | down | Irx3 |
| ENSMUSG00000031877 | 5.33241 | 1.49752 | 1.832216 | 8.87E-11 | 5.53E-09 | up | Ces2g |
| ENSMUSG00000032548 | 8.99037 | 27.9016 | -1.63389 | 1.08E-10 | 6.67E-09 | down | Slco2a1 |
| ENSMUSG00000034266 | 0.73818 | 8.87504 | -3.58771 | 1.21E-10 | 7.46E-09 | down | Batf |
| ENSMUSG00000038775 | 1.64145 | 6.49781 | -1.98498 | 1.22E-10 | 7.51E-09 | down | Vill |
| ENSMUSG00000026576 | 10.418 | 32.1566 | -1.62603 | 1.45E-10 | 8.86E-09 | down | Atp1b1 |
| ENSMUSG00000006356 | 211.314 | 101.456 | 1.058534 | 1.53E-10 | 9.27E-09 | up | Crip2 |
| ENSMUSG00000034910 | 1.89308 | 7.00599 | -1.88786 | 1.56E-10 | 9.41E-09 | down | Pygo1 |
| ENSMUSG00000028399 | 9.34043 | 27.1697 | -1.54044 | 1.59E-10 | 9.54E-09 | down | Ptprd |
| ENSMUSG00000041417 | 6.78066 | 19.6478 | -1.53487 | 1.63E-10 | 9.77E-09 | down | Pik3r1 |
| ENSMUSG00000021327 | 16.1981 | 45.9291 | -1.50358 | 1.65E-10 | 9.85E-09 | down | Zkscan3 |
| ENSMUSG00000024548 | 2.00063 | 7.15396 | -1.83829 | 1.80E-10 | 1.07E-08 | down | Setbp1 |
| ENSMUSG00000105703 | 5.41364 | 2.10906 | 1.359999 | 1.88E-10 | 1.11E-08 | up | Gm43305 |
| ENSMUSG00000023885 | 152.009 | 356.342 | -1.22911 | 2.15E-10 | 1.27E-08 | down | Thbs2 |
| ENSMUSG00000020312 | 11.6211 | 33.5888 | -1.53124 | 2.23E-10 | 1.31E-08 | down | Shc2 |
| ENSMUSG00000113525 | 13.363 | 37.4405 | -1.48635 | 2.25E-10 | 1.32E-08 | down | AC154550.1 |
| ENSMUSG00000059401 | 1.29643 | 5.73541 | -2.14536 | 2.28E-10 | 1.33E-08 | down | Mamld1 |
| ENSMUSG00000043587 | 24.1061 | 77.6573 | -1.68772 | 2.50E-10 | 1.45E-08 | down | Pxylp1 |
| ENSMUSG00000042082 | 31.3271 | 76.52 | -1.28842 | 2.76E-10 | 1.60E-08 | down | Arsb |
| ENSMUSG00000041189 | 16.3095 | 7.29344 | 1.161046 | 2.77E-10 | 1.60E-08 | up | Chrnb1 |
| ENSMUSG00000022037 | 5.08827 | 18.4975 | -1.86208 | 2.98E-10 | 1.71E-08 | down | Clu |
| ENSMUSG00000037621 | 19.791 | 65.1838 | -1.71967 | 3.00E-10 | 1.71E-08 | down | Atoh8 |
| ENSMUSG00000074671 | 5.70861 | 1.75175 | 1.704344 | 2.99E-10 | 1.71E-08 | up | Tspyl3 |
| ENSMUSG00000036334 | 1.759 | 6.23055 | -1.8246 | 3.13E-10 | 1.78E-08 | down | Igsf10 |
| ENSMUSG00000013236 | 28.5108 | 67.5921 | -1.24534 | 3.84E-10 | 2.18E-08 | down | Ptprs |
| ENSMUSG00000024521 | 106.987 | 48.9939 | 1.126764 | 3.97E-10 | 2.25E-08 | up | Pmaip1 |
| ENSMUSG00000027656 | 9.32399 | 29.3363 | -1.65366 | 4.76E-10 | 2.67E-08 | down | Wisp2 |
| ENSMUSG00000053332 | 578.719 | 277.705 | 1.059308 | 4.77E-10 | 2.67E-08 | up | Gas5 |
| ENSMUSG00000030074 | 9.77361 | 21.7176 | -1.1519 | 4.93E-10 | 2.75E-08 | down | Gxylt2 |
| ENSMUSG00000021665 | 65.3316 | 170.216 | -1.38151 | 5.14E-10 | 2.86E-08 | down | Hexb |
| ENSMUSG00000031558 | 9.5252 | 28.5926 | -1.58582 | 5.29E-10 | 2.93E-08 | down | Slit2 |
| ENSMUSG00000046324 | 15.8756 | 45.7792 | -1.52788 | 5.30E-10 | 2.93E-08 | down | Ermp1 |
| ENSMUSG00000025810 | 11.4652 | 30.2012 | -1.39734 | 5.57E-10 | 3.07E-08 | down | Nrp1 |
| ENSMUSG00000021614 | 11.2109 | 36.1162 | -1.68775 | 5.81E-10 | 3.19E-08 | down | Vcan |
| ENSMUSG00000015766 | 22.1496 | 69.0211 | -1.63975 | 6.01E-10 | 3.29E-08 | down | Eps8 |
| ENSMUSG00000027848 | 5.18911 | 27.3076 | -2.39574 | 6.27E-10 | 3.42E-08 | down | Olfml3 |
| ENSMUSG00000030930 | 4.10423 | 12.261 | -1.5789 | 7.92E-10 | 4.25E-08 | down | Chst15 |
| ENSMUSG00000056091 | 17.7785 | 48.4972 | -1.44777 | 8.13E-10 | 4.35E-08 | down | St3gal5 |
| ENSMUSG00000069171 | 1.8246 | 8.02867 | -2.13758 | 8.81E-10 | 4.67E-08 | down | Nr2f1 |
| ENSMUSG00000032062 | 0.10372 | 5.02943 | -5.59959 | 9.97E-10 | 5.24E-08 | down | 2310030G06Rik |
| ENSMUSG00000022438 | 27.1844 | 66.0293 | -1.28033 | 1.03E-09 | 5.37E-08 | down | Parvb |
| ENSMUSG00000009772 | 11.5487 | 5.05022 | 1.193307 | 1.03E-09 | 5.37E-08 | up | Nuak2 |
| ENSMUSG00000057969 | 3.9239 | 13.0282 | -1.73128 | 1.05E-09 | 5.45E-08 | down | Sema3b |
| ENSMUSG00000022223 | 11.1554 | 35.6786 | -1.67731 | 1.20E-09 | 6.19E-08 | down | Sdr39u1 |
| ENSMUSG00000004655 | 0.97779 | 6.16536 | -2.65659 | 1.37E-09 | 7.01E-08 | down | Aqp1 |
| ENSMUSG00000060969 | 1.4308 | 7.08095 | -2.30712 | 1.53E-09 | 7.80E-08 | down | Irx1 |
| ENSMUSG00000045312 | 21.7548 | 54.1903 | -1.3167 | 1.56E-09 | 7.91E-08 | down | Lhfpl2 |
| ENSMUSG00000039578 | 1.47712 | 7.08185 | -2.26134 | 1.60E-09 | 8.10E-08 | down | Ccser1 |
| ENSMUSG00000032128 | 11.3238 | 3.11145 | 1.863701 | 2.06E-09 | 1.02E-07 | up | Robo3 |
| ENSMUSG00000029864 | 0.82206 | 8.83613 | -3.4261 | 2.18E-09 | 1.08E-07 | down | Gstk1 |
| ENSMUSG00000038375 | 17.3623 | 46.9366 | -1.43475 | 2.20E-09 | 1.09E-07 | down | Trp53inp2 |
| ENSMUSG00000030761 | 5.21305 | 12.7727 | -1.29287 | 2.21E-09 | 1.09E-07 | down | Myo7a |
| ENSMUSG00000036006 | 1.48431 | 6.71676 | -2.17797 | 2.40E-09 | 1.18E-07 | down | Fam65b |
| ENSMUSG00000029718 | 179.253 | 468.553 | -1.38621 | 2.41E-09 | 1.18E-07 | down | Pcolce |
| ENSMUSG00000024924 | 10.9848 | 29.4358 | -1.42207 | 2.95E-09 | 1.43E-07 | down | Vldlr |
| ENSMUSG00000041351 | 16.9818 | 47.6664 | -1.48898 | 2.98E-09 | 1.44E-07 | down | Rap1gap |
| ENSMUSG00000037362 | 10.9371 | 29.8298 | -1.44752 | 2.99E-09 | 1.44E-07 | down | Nov |
| ENSMUSG00000039485 | 4.52699 | 14.6805 | -1.69728 | 3.16E-09 | 1.52E-07 | down | Tspyl4 |
| ENSMUSG00000060380 | 1.63347 | 9.85619 | -2.59309 | 3.26E-09 | 1.56E-07 | down | C030014I23Rik |
| ENSMUSG00000030319 | 5.35006 | 14.4643 | -1.43487 | 3.50E-09 | 1.67E-07 | down | Cand2 |
| ENSMUSG00000030287 | 4.00818 | 10.3591 | -1.36988 | 4.12E-09 | 1.94E-07 | down | Itpr2 |
| ENSMUSG00000003617 | 4.14681 | 12.8235 | -1.62872 | 4.21E-09 | 1.98E-07 | down | Cp |
| ENSMUSG00000031750 | 14.4886 | 3.74462 | 1.95203 | 4.23E-09 | 1.98E-07 | up | Il34 |
| ENSMUSG00000029659 | 4.81506 | 15.3268 | -1.67043 | 4.67E-09 | 2.17E-07 | down | Medag |
| ENSMUSG00000026640 | 4.6954 | 11.945 | -1.34709 | 4.69E-09 | 2.17E-07 | down | Plxna2 |
| ENSMUSG00000112980 | 3.90563 | 13.7265 | -1.81333 | 8.10E-09 | 3.72E-07 | down | CT030173.1 |
| ENSMUSG00000030255 | 6.43769 | 17.5389 | -1.44594 | 8.70E-09 | 3.98E-07 | down | Sspn |
| ENSMUSG00000003992 | 3.91067 | 15.8542 | -2.01938 | 9.05E-09 | 4.12E-07 | down | Ssbp2 |
| ENSMUSG00000052534 | 10.9722 | 26.5633 | -1.27558 | 9.18E-09 | 4.16E-07 | down | Pbx1 |
| ENSMUSG00000021950 | 28.8519 | 14.1809 | 1.024718 | 9.17E-09 | 4.16E-07 | up | Anxa8 |
| ENSMUSG00000051331 | 1.45735 | 6.84861 | -2.23247 | 9.63E-09 | 4.34E-07 | down | Cacna1c |
| ENSMUSG00000028776 | 13.1708 | 39.8016 | -1.59548 | 1.03E-08 | 4.62E-07 | down | Tinagl1 |
| ENSMUSG00000052688 | 6.21919 | 14.3015 | -1.20136 | 1.08E-08 | 4.79E-07 | down | Rab7b |
| ENSMUSG00000079164 | 1.08298 | 5.67401 | -2.38937 | 1.18E-08 | 5.21E-07 | down | Tlr5 |
| ENSMUSG00000034334 | 0.83395 | 6.64532 | -2.9943 | 1.19E-08 | 5.24E-07 | down | Fam151b |
| ENSMUSG00000025432 | 12.518 | 36.2616 | -1.53444 | 1.30E-08 | 5.72E-07 | down | Avil |
| ENSMUSG00000029096 | 1.25162 | 8.28423 | -2.72657 | 1.37E-08 | 5.93E-07 | down | Htra3 |
| ENSMUSG00000038776 | 84.8573 | 208.152 | -1.29453 | 1.42E-08 | 6.10E-07 | down | Ephx1 |
| ENSMUSG00000034460 | 3.7366 | 10.5205 | -1.49341 | 1.53E-08 | 6.57E-07 | down | Six4 |
| ENSMUSG00000020674 | 25.1776 | 58.5681 | -1.21797 | 1.88E-08 | 7.97E-07 | down | Pxdn |
| ENSMUSG00000042428 | 4.14851 | 11.645 | -1.48904 | 2.00E-08 | 8.42E-07 | down | Mgat3 |
| ENSMUSG00000078915 | 54.7256 | 26.7423 | 1.033091 | 2.20E-08 | 9.13E-07 | up | Hsp25-ps1 |
| ENSMUSG00000029814 | 1.83251 | 6.67203 | -1.86431 | 2.32E-08 | 9.60E-07 | down | Igf2bp3 |
| ENSMUSG00000000957 | 142.073 | 313.075 | -1.13988 | 2.57E-08 | 1.06E-06 | down | Mmp14 |
| ENSMUSG00000075394 | 7.97462 | 23.6213 | -1.5666 | 2.65E-08 | 1.09E-06 | down | Hoxc4 |
| ENSMUSG00000026748 | 7.0423 | 17.0778 | -1.27801 | 2.91E-08 | 1.18E-06 | down | Plxdc2 |
| ENSMUSG00000028111 | 35.4494 | 88.8682 | -1.32591 | 3.03E-08 | 1.22E-06 | down | Ctsk |
| ENSMUSG00000029673 | 1.63605 | 7.33357 | -2.1643 | 3.14E-08 | 1.26E-06 | down | Auts2 |
| ENSMUSG00000013846 | 7.8982 | 21.1434 | -1.42061 | 3.27E-08 | 1.31E-06 | down | St3gal1 |
| ENSMUSG00000025969 | 102.696 | 221.833 | -1.1111 | 3.33E-08 | 1.33E-06 | down | Nrp2 |
| ENSMUSG00000022754 | 5.1106 | 15.5471 | -1.60508 | 3.38E-08 | 1.35E-06 | down | Tmem45a |
| ENSMUSG00000015709 | 2.68962 | 7.6985 | -1.51718 | 3.61E-08 | 1.43E-06 | down | Arnt2 |
| ENSMUSG00000031538 | 8.88793 | 24.2942 | -1.45069 | 3.58E-08 | 1.43E-06 | down | Plat |
| ENSMUSG00000039518 | 1.62938 | 7.43074 | -2.18919 | 3.70E-08 | 1.46E-06 | down | Cdsn |
| ENSMUSG00000029370 | 5.88263 | 1.98924 | 1.564244 | 3.71E-08 | 1.46E-06 | up | Rassf6 |
| ENSMUSG00000055538 | 39.9632 | 88.7684 | -1.15137 | 4.32E-08 | 1.70E-06 | down | Zcchc24 |
| ENSMUSG00000029019 | 7.89066 | 1.05932 | 2.897005 | 4.67E-08 | 1.81E-06 | up | Nppb |
| ENSMUSG00000027130 | 10.6617 | 25.4644 | -1.25604 | 4.74E-08 | 1.84E-06 | down | Slc12a6 |
| ENSMUSG00000020638 | 2.54942 | 8.68705 | -1.7687 | 5.14E-08 | 1.98E-06 | down | Cmpk2 |
| ENSMUSG00000020241 | 20.4759 | 47.2243 | -1.2056 | 5.14E-08 | 1.98E-06 | down | Col6a2 |
| ENSMUSG00000028164 | 14.5877 | 34.8934 | -1.2582 | 5.35E-08 | 2.05E-06 | down | Manba |
| ENSMUSG00000097769 | 45.3312 | 22.0548 | 1.039408 | 5.57E-08 | 2.12E-06 | up | Snhg4 |
| ENSMUSG00000002997 | 12.1571 | 36.6329 | -1.59134 | 5.89E-08 | 2.23E-06 | down | Prkar2b |
| ENSMUSG00000027750 | 155.295 | 14.3766 | 3.43322 | 5.89E-08 | 2.23E-06 | up | Postn |
| ENSMUSG00000023367 | 22.2903 | 78.0987 | -1.80888 | 6.35E-08 | 2.39E-06 | down | Tmem176a |
| ENSMUSG00000017491 | 2.75558 | 8.88411 | -1.68887 | 6.63E-08 | 2.48E-06 | down | Rarb |
| ENSMUSG00000032625 | 2.41386 | 5.29855 | -1.13425 | 6.64E-08 | 2.48E-06 | down | Thsd7a |
| ENSMUSG00000111977 | 1.07038 | 7.60508 | -2.82884 | 7.52E-08 | 2.77E-06 | down | AC132265.1 |
| ENSMUSG00000035954 | 2.72286 | 8.96094 | -1.71852 | 8.27E-08 | 3.04E-06 | down | Dock4 |
| ENSMUSG00000022905 | 21.1582 | 42.7647 | -1.01521 | 8.68E-08 | 3.18E-06 | down | Kpna1 |
| ENSMUSG00000029810 | 39.1556 | 100.166 | -1.3551 | 8.79E-08 | 3.21E-06 | down | Tmem176b |
| ENSMUSG00000030785 | 5.88971 | 25.3419 | -2.10526 | 9.47E-08 | 3.45E-06 | down | Cox6a2 |
| ENSMUSG00000003849 | 10.0862 | 29.0592 | -1.52661 | 9.68E-08 | 3.51E-06 | down | Nqo1 |
| ENSMUSG00000026925 | 12.365 | 28.5725 | -1.20837 | 9.95E-08 | 3.59E-06 | down | Inpp5e |
| ENSMUSG00000028793 | 96.6231 | 39.69 | 1.283592 | 9.94E-08 | 3.59E-06 | up | Rnf19b |
| ENSMUSG00000037086 | 6.06071 | 1.26953 | 2.255191 | 1.11E-07 | 3.98E-06 | up | Prr32 |
| ENSMUSG00000014602 | 1.41952 | 6.08158 | -2.09904 | 1.16E-07 | 4.12E-06 | down | Kif1a |
| ENSMUSG00000033705 | 5.77892 | 13.3337 | -1.2062 | 1.17E-07 | 4.15E-06 | down | Stard9 |
| ENSMUSG00000031886 | 7.67578 | 2.23592 | 1.779442 | 1.22E-07 | 4.33E-06 | up | Ces2e |
| ENSMUSG00000024899 | 2.33817 | 7.32837 | -1.64811 | 1.46E-07 | 5.14E-06 | down | Papss2 |
| ENSMUSG00000024066 | 45.3785 | 101.082 | -1.15544 | 1.56E-07 | 5.48E-06 | down | Xdh |
| ENSMUSG00000075585 | 2.35083 | 6.74157 | -1.51992 | 1.65E-07 | 5.76E-06 | down | 6330403L08Rik |
| ENSMUSG00000071552 | 7.92157 | 1.94162 | 2.028522 | 1.70E-07 | 5.88E-06 | up | Tigit |
| ENSMUSG00000022562 | 5.13987 | 12.0527 | -1.22956 | 1.73E-07 | 6.00E-06 | down | Oplah |
| ENSMUSG00000020256 | 37.624 | 91.1921 | -1.27725 | 1.86E-07 | 6.41E-06 | down | Aldh1l2 |
| ENSMUSG00000027995 | 4.36435 | 12.3536 | -1.5011 | 1.89E-07 | 6.50E-06 | down | Tlr2 |
| ENSMUSG00000029755 | 6.45968 | 20.9332 | -1.69626 | 2.11E-07 | 7.21E-06 | down | Dlx5 |
| ENSMUSG00000021754 | 2.43913 | 7.07694 | -1.53676 | 2.12E-07 | 7.25E-06 | down | Map3k1 |
| ENSMUSG00000039787 | 20.6547 | 44.3845 | -1.10358 | 2.17E-07 | 7.37E-06 | down | Cercam |
| ENSMUSG00000020227 | 3.08472 | 8.65311 | -1.48808 | 2.23E-07 | 7.54E-06 | down | Irak3 |
| ENSMUSG00000036867 | 16.541 | 41.1404 | -1.31451 | 2.30E-07 | 7.72E-06 | down | Smad6 |
| ENSMUSG00000036565 | 28.6864 | 62.2418 | -1.11752 | 2.36E-07 | 7.93E-06 | down | Ttyh3 |
| ENSMUSG00000028358 | 2.30477 | 6.06446 | -1.39576 | 2.63E-07 | 8.78E-06 | down | Zfp618 |
| ENSMUSG00000034586 | 6.29363 | 14.8225 | -1.23582 | 2.66E-07 | 8.85E-06 | down | Hid1 |
| ENSMUSG00000020695 | 45.188 | 93.5851 | -1.05034 | 2.66E-07 | 8.86E-06 | down | Mrc2 |
| ENSMUSG00000040260 | 3.60337 | 9.25131 | -1.36031 | 2.79E-07 | 9.23E-06 | down | Daam2 |
| ENSMUSG00000050199 | 7.1388 | 17.3855 | -1.28413 | 2.88E-07 | 9.49E-06 | down | Lgr4 |
| ENSMUSG00000066026 | 8.75976 | 29.0242 | -1.72829 | 2.92E-07 | 9.61E-06 | down | Dhrs3 |
| ENSMUSG00000060550 | 8.38494 | 19.2877 | -1.20181 | 3.05E-07 | 1.00E-05 | down | H2-Q7 |
| ENSMUSG00000056947 | 1.45939 | 6.38764 | -2.12992 | 3.08E-07 | 1.01E-05 | down | Mab21l1 |
| ENSMUSG00000021508 | 11.298 | 33.3349 | -1.56096 | 3.13E-07 | 1.02E-05 | down | Cxcl14 |
| ENSMUSG00000025950 | 13.2835 | 31.7 | -1.25485 | 3.13E-07 | 1.02E-05 | down | Idh1 |
| ENSMUSG00000111511 | 6.06101 | 1.11627 | 2.440876 | 3.14E-07 | 1.02E-05 | up | AC160966.2 |
| ENSMUSG00000038296 | 7.42189 | 21.0693 | -1.50528 | 3.16E-07 | 1.03E-05 | down | Galnt18 |
| ENSMUSG00000041734 | 64.894 | 136.48 | -1.07253 | 3.19E-07 | 1.04E-05 | down | Kirrel |
| ENSMUSG00000025453 | 3.24328 | 10.599 | -1.7084 | 3.50E-07 | 1.13E-05 | down | Nnt |
| ENSMUSG00000017631 | 30.105 | 69.4877 | -1.20676 | 3.75E-07 | 1.20E-05 | down | Abr |
| ENSMUSG00000073409 | 5.70625 | 15.7285 | -1.46277 | 4.58E-07 | 1.45E-05 | down | H2-Q6 |
| ENSMUSG00000029754 | 0.91715 | 6.08318 | -2.72959 | 4.76E-07 | 1.50E-05 | down | Dlx6 |
| ENSMUSG00000002980 | 2.17338 | 8.1893 | -1.9138 | 5.15E-07 | 1.62E-05 | down | Bcam |
| ENSMUSG00000008734 | 3.88815 | 11.4778 | -1.56169 | 5.23E-07 | 1.63E-05 | down | Gprc5b |
| ENSMUSG00000038213 | 4.43944 | 13.5743 | -1.61243 | 6.41E-07 | 1.99E-05 | down | Tapbpl |
| ENSMUSG00000034761 | 24.9176 | 54.4532 | -1.12785 | 6.55E-07 | 2.02E-05 | down | Map4k5 |
| ENSMUSG00000024053 | 21.9073 | 50.6182 | -1.20824 | 6.59E-07 | 2.03E-05 | down | Emilin2 |
| ENSMUSG00000055799 | 13.2552 | 32.5371 | -1.29553 | 6.63E-07 | 2.04E-05 | down | Tcf7l1 |
| ENSMUSG00000011148 | 7.85754 | 21.5581 | -1.45608 | 6.86E-07 | 2.10E-05 | down | Adssl1 |
| ENSMUSG00000035105 | 3.62497 | 11.8501 | -1.70886 | 7.87E-07 | 2.40E-05 | down | Egln3 |
| ENSMUSG00000026664 | 19.0004 | 45.027 | -1.24476 | 7.91E-07 | 2.41E-05 | down | Phyh |
| ENSMUSG00000058571 | 7.56007 | 16.5852 | -1.13342 | 8.15E-07 | 2.47E-05 | down | Gpc6 |
| ENSMUSG00000067212 | 6.56399 | 23.4804 | -1.83881 | 8.89E-07 | 2.66E-05 | down | H2-T23 |
| ENSMUSG00000029468 | 5.88083 | 2.70419 | 1.120825 | 9.03E-07 | 2.70E-05 | up | P2rx7 |
| ENSMUSG00000003363 | 39.0384 | 83.2009 | -1.0917 | 9.55E-07 | 2.84E-05 | down | Pld3 |
| ENSMUSG00000049804 | 3.91182 | 10.4709 | -1.42047 | 1.03E-06 | 3.05E-05 | down | Armcx4 |
| ENSMUSG00000004885 | 15.104 | 48.0975 | -1.67103 | 1.06E-06 | 3.11E-05 | down | Crabp2 |
| ENSMUSG00000038712 | 30.8135 | 68.0594 | -1.14323 | 1.21E-06 | 3.55E-05 | down | Fam63a |
| ENSMUSG00000018819 | 29.1464 | 13.1411 | 1.149229 | 1.23E-06 | 3.61E-05 | up | Lsp1 |
| ENSMUSG00000025366 | 56.0326 | 116.047 | -1.05038 | 1.24E-06 | 3.62E-05 | down | Esyt1 |
| ENSMUSG00000037254 | 3.15801 | 9.69118 | -1.61766 | 1.37E-06 | 3.97E-05 | down | Itih2 |
| ENSMUSG00000029816 | 151.868 | 309.727 | -1.02817 | 1.39E-06 | 4.01E-05 | down | Gpnmb |
| ENSMUSG00000084762 | 5.43189 | 0.0001 | 15.72917 | 1.39E-06 | 4.01E-05 | up | Platr3 |
| ENSMUSG00000027315 | 4.55134 | 11.9605 | -1.39391 | 1.69E-06 | 4.80E-05 | down | Spint1 |
| ENSMUSG00000041132 | 1.9442 | 7.88436 | -2.01982 | 1.82E-06 | 5.09E-05 | down | N4bp2l1 |
| ENSMUSG00000051022 | 9.85989 | 32.9159 | -1.73914 | 1.87E-06 | 5.21E-05 | down | Hs3st1 |
| ENSMUSG00000009418 | 15.1867 | 33.9327 | -1.15987 | 1.88E-06 | 5.22E-05 | down | Nav1 |
| ENSMUSG00000094365 | 11.0233 | 0.71122 | 3.954125 | 1.87E-06 | 5.22E-05 | up | Gm21982 |
| ENSMUSG00000006205 | 35.065 | 81.0605 | -1.20897 | 1.93E-06 | 5.35E-05 | down | Htra1 |
| ENSMUSG00000027954 | 6.80342 | 19.5568 | -1.52334 | 2.12E-06 | 5.85E-05 | down | Efna1 |
| ENSMUSG00000041112 | 7.76699 | 17.3633 | -1.16062 | 2.25E-06 | 6.13E-05 | down | Elmo1 |
| ENSMUSG00000019866 | 2.95629 | 7.34108 | -1.31221 | 2.25E-06 | 6.14E-05 | down | Aim1 |
| ENSMUSG00000011837 | 50.4232 | 24.1049 | 1.06476 | 2.34E-06 | 6.37E-05 | up | Snapc2 |
| ENSMUSG00000042078 | 5.44851 | 2.02421 | 1.428504 | 2.39E-06 | 6.47E-05 | up | Svop |
| ENSMUSG00000040033 | 15.9582 | 35.9428 | -1.1714 | 2.40E-06 | 6.50E-05 | down | Stat2 |
| ENSMUSG00000006586 | 5.08403 | 2.50756 | 1.019691 | 2.50E-06 | 6.73E-05 | up | Runx1t1 |
| ENSMUSG00000008540 | 8.09946 | 24.3161 | -1.58601 | 2.51E-06 | 6.74E-05 | down | Mgst1 |
| ENSMUSG00000031378 | 10.4736 | 24.434 | -1.22214 | 2.91E-06 | 7.80E-05 | down | Abcd1 |
| ENSMUSG00000029334 | 66.7653 | 32.777 | 1.026417 | 2.95E-06 | 7.88E-05 | up | Prkg2 |
| ENSMUSG00000059555 | 11.5878 | 28.7832 | -1.31263 | 2.97E-06 | 7.92E-05 | down | Tor4a |
| ENSMUSG00000050910 | 39.9433 | 81.6263 | -1.03108 | 2.99E-06 | 7.96E-05 | down | Cdr2l |
| ENSMUSG00000030257 | 8.33855 | 17.2205 | -1.04626 | 3.12E-06 | 8.28E-05 | down | Srgap3 |
| ENSMUSG00000052504 | 0.02768 | 7.23614 | -8.03039 | 3.13E-06 | 8.29E-05 | down | Epha3 |
| ENSMUSG00000028965 | 0.60495 | 7.46599 | -3.62545 | 3.18E-06 | 8.41E-05 | down | Tnfrsf9 |
| ENSMUSG00000019872 | 2.18453 | 7.8572 | -1.84669 | 3.24E-06 | 8.54E-05 | down | Smpdl3a |
| ENSMUSG00000096210 | 234.202 | 532.422 | -1.18482 | 3.32E-06 | 8.74E-05 | down | H1f0 |
| ENSMUSG00000074457 | 29.1073 | 74.4207 | -1.35432 | 3.40E-06 | 8.90E-05 | down | S100a16 |
| ENSMUSG00000051316 | 5.19811 | 2.50128 | 1.055318 | 3.46E-06 | 9.01E-05 | up | Taf7 |
| ENSMUSG00000064359 | 4.99052 | 64.0421 | -3.68176 | 3.47E-06 | 9.04E-05 | down | mt-Tg |
| ENSMUSG00000074364 | 55.032 | 113.709 | -1.04701 | 3.67E-06 | 9.53E-05 | down | Ehd2 |
| ENSMUSG00000064043 | 3.69875 | 9.44336 | -1.35226 | 3.92E-06 | 0.000102 | down | Trerf1 |
| ENSMUSG00000071064 | 5.65544 | 13.0206 | -1.20309 | 4.27E-06 | 0.00011 | down | Zfp827 |
| ENSMUSG00000035437 | 19.1077 | 38.2735 | -1.00219 | 4.31E-06 | 0.000111 | down | Rabgap1 |
| ENSMUSG00000002006 | 3.21036 | 8.38919 | -1.3858 | 4.67E-06 | 0.000119 | down | Pdzd4 |
| ENSMUSG00000098470 | 3.13912 | 9.55579 | -1.60601 | 4.78E-06 | 0.000122 | down | C1rb |
| ENSMUSG00000021280 | 8.73752 | 26.6723 | -1.61005 | 4.96E-06 | 0.000126 | down | Exoc3l4 |
| ENSMUSG00000035547 | 3.26675 | 8.43615 | -1.36873 | 5.16E-06 | 0.000131 | down | Capn5 |
| ENSMUSG00000021253 | 15.9444 | 34.4835 | -1.11286 | 5.39E-06 | 0.000136 | down | Tgfb3 |
| ENSMUSG00000028278 | 2.79161 | 7.92431 | -1.50519 | 5.51E-06 | 0.000139 | down | Rragd |
| ENSMUSG00000078612 | 5.5377 | 2.48402 | 1.156612 | 5.57E-06 | 0.00014 | up | 1700024P16Rik |
| ENSMUSG00000035914 | 18.2194 | 38.3174 | -1.07253 | 5.69E-06 | 0.000142 | down | Cd276 |
| ENSMUSG00000026482 | 18.5049 | 39.679 | -1.10047 | 5.81E-06 | 0.000144 | down | Rgl1 |
| ENSMUSG00000039476 | 77.3993 | 168.866 | -1.12548 | 5.86E-06 | 0.000145 | down | Prrx2 |
| ENSMUSG00000032232 | 26.5759 | 56.2068 | -1.08063 | 6.27E-06 | 0.000154 | down | Cgnl1 |
| ENSMUSG00000089762 | 15.0497 | 33.6359 | -1.16027 | 6.48E-06 | 0.000158 | down | Ier5l |
| ENSMUSG00000032363 | 2.13212 | 5.03693 | -1.24026 | 6.62E-06 | 0.000161 | down | Adamts7 |
| ENSMUSG00000049420 | 1.63367 | 5.47557 | -1.7449 | 7.21E-06 | 0.000173 | down | Tmem200a |
| ENSMUSG00000027227 | 31.6002 | 70.359 | -1.1548 | 7.21E-06 | 0.000173 | down | Sord |
| ENSMUSG00000000901 | 32.8692 | 71.8663 | -1.12858 | 7.27E-06 | 0.000175 | down | Mmp11 |
| ENSMUSG00000062300 | 11.8422 | 28.6457 | -1.27438 | 8.43E-06 | 0.000199 | down | Nectin2 |
| ENSMUSG00000041075 | 17.2973 | 35.1302 | -1.02217 | 8.54E-06 | 0.000201 | down | Fzd7 |
| ENSMUSG00000016552 | 6.41337 | 14.8229 | -1.20867 | 9.24E-06 | 0.000216 | down | Foxred2 |
| ENSMUSG00000102813 | 11.3307 | 3.26373 | 1.795648 | 1.01E-05 | 0.000234 | up | Gm37795 |
| ENSMUSG00000034997 | 6.41273 | 2.32838 | 1.461613 | 1.04E-05 | 0.00024 | up | Htr2a |
| ENSMUSG00000040430 | 4.49897 | 10.7118 | -1.25154 | 1.06E-05 | 0.000243 | down | Pitpnc1 |
| ENSMUSG00000021190 | 76.6709 | 156.157 | -1.02625 | 1.07E-05 | 0.000247 | down | Lgmn |
| ENSMUSG00000028476 | 4.96717 | 12.2603 | -1.30349 | 1.08E-05 | 0.000247 | down | Reck |
| ENSMUSG00000002603 | 41.8475 | 87.4875 | -1.06394 | 1.09E-05 | 0.000249 | down | Tgfb1 |
| ENSMUSG00000027996 | 33.9306 | 75.1087 | -1.14639 | 1.10E-05 | 0.000251 | down | Sfrp2 |
| ENSMUSG00000061451 | 5.59142 | 13.2252 | -1.242 | 1.11E-05 | 0.000253 | down | Tmem151a |
| ENSMUSG00000024900 | 21.5185 | 45.3388 | -1.07517 | 1.18E-05 | 0.000264 | down | Cpt1a |
| ENSMUSG00000054263 | 5.25537 | 11.4724 | -1.12631 | 1.19E-05 | 0.000266 | down | Lifr |
| ENSMUSG00000009614 | 19.7765 | 9.63201 | 1.037878 | 1.22E-05 | 0.000273 | up | Sardh |
| ENSMUSG00000000693 | 95.0352 | 190.708 | -1.00483 | 1.31E-05 | 0.00029 | down | Loxl3 |
| ENSMUSG00000022995 | 40.7271 | 82.493 | -1.01828 | 1.35E-05 | 0.000297 | down | Enah |
| ENSMUSG00000034771 | 7.7077 | 17.4229 | -1.17662 | 1.35E-05 | 0.000297 | down | Tle2 |
| ENSMUSG00000020964 | 19.2853 | 39.6426 | -1.03955 | 1.38E-05 | 0.000303 | down | Sel1l |
| ENSMUSG00000024866 | 13.7832 | 6.34954 | 1.118185 | 1.40E-05 | 0.000306 | up | Acy3 |
| ENSMUSG00000020647 | 5.36301 | 17.6357 | -1.71739 | 1.42E-05 | 0.000309 | down | Ncoa1 |
| ENSMUSG00000036103 | 64.6803 | 136.55 | -1.07803 | 1.44E-05 | 0.000314 | down | Colec12 |
| ENSMUSG00000070942 | 2.99634 | 8.07125 | -1.42959 | 1.58E-05 | 0.000342 | down | Il1rl2 |
| ENSMUSG00000057777 | 11.3024 | 24.9421 | -1.14195 | 1.62E-05 | 0.00035 | down | Mab21l2 |
| ENSMUSG00000106847 | 3.00424 | 7.28835 | -1.27859 | 1.76E-05 | 0.000376 | down | Peg13 |
| ENSMUSG00000074794 | 6.18101 | 15.3829 | -1.31541 | 1.80E-05 | 0.000384 | down | Arrdc3 |
| ENSMUSG00000028121 | 5.22602 | 12.9389 | -1.30793 | 1.89E-05 | 0.0004 | down | Bcar3 |
| ENSMUSG00000034220 | 51.2165 | 107.424 | -1.06863 | 1.90E-05 | 0.000402 | down | Gpc1 |
| ENSMUSG00000079685 | 6.70121 | 15.6737 | -1.22585 | 1.96E-05 | 0.000413 | down | Ulbp1 |
| ENSMUSG00000026072 | 3.87619 | 9.23636 | -1.25268 | 2.00E-05 | 0.000421 | down | Il1r1 |
| ENSMUSG00000018417 | 26.5884 | 56.8835 | -1.09721 | 2.25E-05 | 0.000469 | down | Myo1b |
| ENSMUSG00000025854 | 20.0945 | 43.4044 | -1.11104 | 2.29E-05 | 0.000476 | down | Fam20c |
| ENSMUSG00000027833 | 36.2233 | 73.3314 | -1.01751 | 2.34E-05 | 0.000484 | down | Shox2 |
| ENSMUSG00000027796 | 14.6078 | 29.5257 | -1.01524 | 2.41E-05 | 0.000498 | down | Smad9 |
| ENSMUSG00000036155 | 9.74297 | 20.1632 | -1.04929 | 2.47E-05 | 0.000508 | down | Mgat5 |
| ENSMUSG00000078502 | 10.6001 | 4.51424 | 1.231525 | 2.56E-05 | 0.000524 | up | Gm13212 |
| ENSMUSG00000032322 | 3.098 | 7.88772 | -1.34827 | 2.65E-05 | 0.000542 | down | Pstpip1 |
| ENSMUSG00000026571 | 10.4389 | 26.8133 | -1.36098 | 2.88E-05 | 0.000579 | down | Dcaf6 |
| ENSMUSG00000029561 | 13.2313 | 36.53 | -1.46513 | 2.95E-05 | 0.000591 | down | Oasl2 |
| ENSMUSG00000022353 | 6.89916 | 14.5187 | -1.07342 | 2.99E-05 | 0.000598 | down | Mtss1 |
| ENSMUSG00000030638 | 1.18612 | 5.85773 | -2.30409 | 3.23E-05 | 0.000639 | down | Sh3gl3 |
| ENSMUSG00000027335 | 2.36152 | 7.99213 | -1.75886 | 3.25E-05 | 0.000642 | down | Adra1d |
| ENSMUSG00000027239 | 0.37037 | 5.40119 | -3.86624 | 3.35E-05 | 0.000657 | down | Mdk |
| ENSMUSG00000041261 | 5.62992 | 12.5282 | -1.154 | 3.38E-05 | 0.000663 | down | Car8 |
| ENSMUSG00000025466 | 11.8584 | 32.3771 | -1.44907 | 3.41E-05 | 0.000667 | down | Fuom |
| ENSMUSG00000031700 | 24.4995 | 50.846 | -1.05338 | 3.41E-05 | 0.000667 | down | Gpt2 |
| ENSMUSG00000074934 | 76.6849 | 167.611 | -1.1281 | 3.44E-05 | 0.000671 | down | Grem1 |
| ENSMUSG00000026932 | 9.57578 | 20.3673 | -1.0888 | 3.67E-05 | 0.000711 | down | Nacc2 |
| ENSMUSG00000029163 | 29.7728 | 60.8263 | -1.0307 | 3.67E-05 | 0.000711 | down | Emilin1 |
| ENSMUSG00000032846 | 8.69328 | 18.0289 | -1.05233 | 3.85E-05 | 0.000742 | down | Zswim6 |
| ENSMUSG00000057329 | 3.75631 | 8.76635 | -1.22266 | 4.13E-05 | 0.000792 | down | Bcl2 |
| ENSMUSG00000018986 | 9.08974 | 4.3415 | 1.066046 | 4.19E-05 | 0.000801 | up | Slfn3 |
| ENSMUSG00000061878 | 9.17643 | 19.6261 | -1.09677 | 4.38E-05 | 0.000829 | down | Sphk1 |
| ENSMUSG00000037035 | 3.41921 | 8.14673 | -1.25256 | 4.43E-05 | 0.000838 | down | Inhbb |
| ENSMUSG00000090399 | 3.17726 | 11.1992 | -1.81755 | 4.52E-05 | 0.000851 | down | Gm38399 |
| ENSMUSG00000020131 | 12.0211 | 5.95976 | 1.012237 | 4.54E-05 | 0.000854 | up | Pcsk4 |
| ENSMUSG00000064138 | 10.0592 | 21.8574 | -1.11961 | 4.57E-05 | 0.000858 | down | Fam172a |
| ENSMUSG00000027797 | 10.6899 | 23.3482 | -1.12707 | 4.76E-05 | 0.000888 | down | Dclk1 |
| ENSMUSG00000096780 | 13.2242 | 29.5641 | -1.16067 | 4.79E-05 | 0.000891 | down | Tmem181b-ps |
| ENSMUSG00000005958 | 5.7877 | 12.8022 | -1.14533 | 4.85E-05 | 0.000901 | down | Ephb3 |
| ENSMUSG00000032202 | 3.06352 | 8.63447 | -1.49492 | 4.96E-05 | 0.000919 | down | Rab27a |
| ENSMUSG00000020576 | 11.1563 | 23.3725 | -1.06695 | 5.25E-05 | 0.000968 | down | Nbas |
| ENSMUSG00000113302 | 5.78009 | 2.6358 | 1.132849 | 5.59E-05 | 0.001022 | up | AC122354.1 |
| ENSMUSG00000052085 | 4.76533 | 10.0409 | -1.07523 | 5.71E-05 | 0.001041 | down | Dock8 |
| ENSMUSG00000026674 | 63.6431 | 144.04 | -1.17839 | 5.73E-05 | 0.001044 | down | Ddr2 |
| ENSMUSG00000042659 | 3.13659 | 8.52513 | -1.44253 | 5.75E-05 | 0.001046 | down | Arrdc4 |
| ENSMUSG00000034109 | 25.8182 | 54.5658 | -1.07961 | 5.78E-05 | 0.00105 | down | Golim4 |
| ENSMUSG00000031216 | 4.58282 | 10.7953 | -1.23609 | 5.83E-05 | 0.001057 | down | Stard8 |
| ENSMUSG00000040234 | 18.4622 | 37.9404 | -1.03916 | 5.96E-05 | 0.001077 | down | Tm7sf3 |
| ENSMUSG00000063838 | 0.94558 | 5.99057 | -2.66343 | 6.04E-05 | 0.00109 | down | 1700027J19Rik |
| ENSMUSG00000044927 | 47.274 | 105.557 | -1.15891 | 6.10E-05 | 0.001098 | down | H1fx |
| ENSMUSG00000021725 | 18.4263 | 37.3045 | -1.01758 | 6.17E-05 | 0.001108 | down | Parp8 |
| ENSMUSG00000027580 | 10.1362 | 20.636 | -1.02565 | 6.33E-05 | 0.001133 | down | Helz2 |
| ENSMUSG00000033585 | 4.49237 | 10.1879 | -1.18131 | 6.46E-05 | 0.001154 | down | Ndn |
| ENSMUSG00000058325 | 17.2884 | 34.727 | -1.00626 | 7.00E-05 | 0.001245 | down | Dock1 |
| ENSMUSG00000045095 | 5.19852 | 11.0238 | -1.08445 | 7.11E-05 | 0.001257 | down | Magi1 |
| ENSMUSG00000112336 | 9.9744 | 34.1369 | -1.77503 | 7.59E-05 | 0.001326 | down | CT030161.2 |
| ENSMUSG00000030748 | 9.14374 | 19.949 | -1.12546 | 7.63E-05 | 0.001333 | down | Il4ra |
| ENSMUSG00000054293 | 1.5424 | 6.64574 | -2.10725 | 7.92E-05 | 0.001367 | down | A630033H20Rik |
| ENSMUSG00000033880 | 74.7315 | 154.199 | -1.045 | 7.94E-05 | 0.001368 | down | Lgals3bp |
| ENSMUSG00000022887 | 15.6008 | 32.3282 | -1.05117 | 7.98E-05 | 0.001369 | down | Masp1 |
| ENSMUSG00000093930 | 14.8034 | 30.2069 | -1.02895 | 7.98E-05 | 0.001369 | down | Hmgcs1 |
| ENSMUSG00000024663 | 4.85208 | 13.1396 | -1.43725 | 8.17E-05 | 0.001401 | down | Rab3il1 |
| ENSMUSG00000024846 | 1.75201 | 5.21924 | -1.57483 | 8.39E-05 | 0.001434 | down | Cst6 |
| ENSMUSG00000001751 | 14.2434 | 30.9697 | -1.12057 | 8.71E-05 | 0.001481 | down | Naglu |
| ENSMUSG00000039765 | 7.69736 | 15.9112 | -1.04761 | 9.00E-05 | 0.001526 | down | Cc2d2a |
| ENSMUSG00000010307 | 25.3067 | 59.2917 | -1.22831 | 9.02E-05 | 0.001527 | down | Tmem86a |
| ENSMUSG00000030020 | 3.942 | 8.52055 | -1.11202 | 9.17E-05 | 0.001549 | down | Prickle2 |
| ENSMUSG00000090290 | 4.86956 | 10.0778 | -1.04932 | 9.43E-05 | 0.001589 | down | Tarbp1 |
| ENSMUSG00000025498 | 4.60321 | 18.3934 | -1.99847 | 9.64E-05 | 0.001617 | down | Irf7 |
| ENSMUSG00000000440 | 14.4451 | 31.4976 | -1.12467 | 9.63E-05 | 0.001617 | down | Pparg |
| ENSMUSG00000057914 | 3.81133 | 9.28736 | -1.28497 | 9.92E-05 | 0.001659 | down | Cacnb2 |
| ENSMUSG00000034401 | 14.7543 | 31.4939 | -1.09393 | 0.000101 | 0.001682 | down | Spata6 |
| ENSMUSG00000060519 | 12.8219 | 27.2112 | -1.0856 | 0.000102 | 0.001696 | down | Tor3a |
| ENSMUSG00000025860 | 14.4482 | 37.3893 | -1.37174 | 0.000105 | 0.001732 | down | Xiap |
| ENSMUSG00000085241 | 114.46 | 53.1903 | 1.105614 | 0.000109 | 0.001785 | up | Snhg3 |
| ENSMUSG00000001657 | 6.94377 | 15.9695 | -1.20153 | 0.000112 | 0.001829 | down | Hoxc8 |
| ENSMUSG00000038235 | 19.2602 | 38.8174 | -1.01108 | 0.000113 | 0.001838 | down | F11r |
| ENSMUSG00000036854 | 25.7813 | 51.6913 | -1.0036 | 0.000114 | 0.001852 | down | Hspb6 |
| ENSMUSG00000022156 | 7.81834 | 2.9725 | 1.395187 | 0.000117 | 0.001887 | up | Gzme |
| ENSMUSG00000071856 | 4.79566 | 10.5263 | -1.13419 | 0.000117 | 0.001894 | down | Mcc |
| ENSMUSG00000093594 | 5.11233 | 2.43611 | 1.069403 | 0.000117 | 0.001894 | up | Gm20707 |
| ENSMUSG00000020641 | 5.34328 | 11.983 | -1.16519 | 0.000121 | 0.00195 | down | Rsad2 |
| ENSMUSG00000042111 | 15.96 | 33.3794 | -1.0645 | 0.000122 | 0.00195 | down | Ccdc115 |
| ENSMUSG00000025504 | 7.60272 | 19.1887 | -1.33567 | 0.000125 | 0.001999 | down | Eps8l2 |
| ENSMUSG00000044548 | 2.38701 | 5.65378 | -1.24402 | 0.000135 | 0.00214 | down | Dact1 |
| ENSMUSG00000035413 | 29.4613 | 60.2092 | -1.03116 | 0.000145 | 0.002255 | down | Tmem98 |
| ENSMUSG00000028760 | 39.1663 | 80.007 | -1.03051 | 0.000147 | 0.002278 | down | Eif4g3 |
| ENSMUSG00000058254 | 3.56214 | 9.71247 | -1.44709 | 0.00015 | 0.002327 | down | Tspan7 |
| ENSMUSG00000032014 | 20.172 | 41.9184 | -1.05523 | 0.000154 | 0.002369 | down | Oaf |
| ENSMUSG00000079560 | 3.30446 | 8.34957 | -1.33729 | 0.000159 | 0.002431 | down | Hoxa3 |
| ENSMUSG00000044216 | 5.05387 | 2.38029 | 1.086253 | 0.000168 | 0.002553 | up | Kcnj4 |
| ENSMUSG00000026471 | 10.7957 | 24.527 | -1.18391 | 0.000172 | 0.002613 | down | Mr1 |
| ENSMUSG00000037211 | 2.82502 | 7.315 | -1.3726 | 0.000177 | 0.002654 | down | Spry1 |
| ENSMUSG00000040105 | 5.36962 | 12.0496 | -1.1661 | 0.000182 | 0.002724 | down | Plpp6 |
| ENSMUSG00000098371 | 61.9626 | 172.428 | -1.47652 | 0.000189 | 0.00281 | down | Gm28037 |
| ENSMUSG00000079018 | 13.7975 | 32.9817 | -1.25726 | 0.000191 | 0.002834 | down | Ly6c1 |
| ENSMUSG00000030107 | 5.54427 | 16.1168 | -1.53949 | 0.000205 | 0.003018 | down | Usp18 |
| ENSMUSG00000062044 | 1.47997 | 5.63388 | -1.92856 | 0.00021 | 0.003087 | down | Lmtk3 |
| ENSMUSG00000042363 | 12.9718 | 26.6197 | -1.03711 | 0.000211 | 0.003091 | down | Lgalsl |
| ENSMUSG00000040714 | 2.45293 | 7.98583 | -1.70294 | 0.000217 | 0.003179 | down | Klc3 |
| ENSMUSG00000018750 | 5.98275 | 18.2577 | -1.60963 | 0.000222 | 0.003234 | down | Zbtb4 |
| ENSMUSG00000007594 | 2.37918 | 6.35175 | -1.41669 | 0.000232 | 0.003368 | down | Hapln4 |
| ENSMUSG00000043671 | 4.79872 | 10.9871 | -1.19509 | 0.000235 | 0.003402 | down | Dpy19l3 |
| ENSMUSG00000103749 | 0.01485 | 12.0676 | -9.66665 | 0.000236 | 0.003407 | down | Pcdhgb5 |
| ENSMUSG00000079499 | 3.59324 | 7.7923 | -1.11677 | 0.000238 | 0.003438 | down | 6530402F18Rik |
| ENSMUSG00000037990 | 3.51195 | 7.80714 | -1.15252 | 0.00024 | 0.003459 | down | Sh3rf3 |
| ENSMUSG00000027890 | 2.36378 | 6.93216 | -1.55221 | 0.000241 | 0.003472 | down | Gstm4 |
| ENSMUSG00000042444 | 6.483 | 14.8456 | -1.1953 | 0.000247 | 0.003542 | down | Fam63b |
| ENSMUSG00000027624 | 43.7429 | 89.9418 | -1.03994 | 0.000251 | 0.003582 | down | Epb41l1 |
| ENSMUSG00000051367 | 14.3182 | 41.499 | -1.53522 | 0.000264 | 0.003758 | down | Six1 |
| ENSMUSG00000062980 | 6.8414 | 13.9517 | -1.02808 | 0.000268 | 0.003804 | down | Cped1 |
| ENSMUSG00000035112 | 2.45837 | 6.64999 | -1.43565 | 0.000276 | 0.003897 | down | Wnk4 |
| ENSMUSG00000005886 | 11.9614 | 24.6161 | -1.04122 | 0.000279 | 0.003924 | down | Ncoa2 |
| ENSMUSG00000062866 | 11.1843 | 22.5159 | -1.00947 | 0.000282 | 0.00396 | down | Phactr2 |
| ENSMUSG00000053175 | 4.52573 | 10.6121 | -1.22949 | 0.000298 | 0.004153 | down | Bcl3 |
| ENSMUSG00000028445 | 5.46134 | 2.09452 | 1.382634 | 0.000305 | 0.004245 | up | Enho |
| ENSMUSG00000003559 | 2.32046 | 7.09289 | -1.61196 | 0.000306 | 0.004256 | down | As3mt |
| ENSMUSG00000034640 | 13.6276 | 27.3709 | -1.00611 | 0.000308 | 0.004261 | down | Tiparp |
| ENSMUSG00000025650 | 2.44938 | 6.23728 | -1.3485 | 0.000312 | 0.004306 | down | Col7a1 |
| ENSMUSG00000070720 | 11.6169 | 25.2969 | -1.12274 | 0.000316 | 0.004343 | down | Tmem200b |
| ENSMUSG00000109523 | 6.46011 | 15.4592 | -1.25883 | 0.000321 | 0.0044 | down | Gdf1 |
| ENSMUSG00000021094 | 16.8186 | 37.8097 | -1.1687 | 0.000332 | 0.00453 | down | Dhrs7 |
| ENSMUSG00000025815 | 2.51655 | 5.09147 | -1.01663 | 0.000333 | 0.004551 | down | Dhtkd1 |
| ENSMUSG00000036904 | 7.96884 | 16.0369 | -1.00895 | 0.000341 | 0.004622 | down | Fzd8 |
| ENSMUSG00000093071 | 54.2134 | 0.383 | 7.145155 | 0.000342 | 0.004629 | up | Gm25951 |
| ENSMUSG00000110206 | 6.17412 | 14.3775 | -1.21951 | 0.000348 | 0.004689 | down | Flt3l |
| ENSMUSG00000036188 | 11.6664 | 23.9234 | -1.03607 | 0.000361 | 0.004837 | down | Ankmy2 |
| ENSMUSG00000087408 | 3.48711 | 7.80587 | -1.16253 | 0.000367 | 0.00489 | down | Cers1 |
| ENSMUSG00000019775 | 2.55901 | 6.36401 | -1.31435 | 0.000369 | 0.004907 | down | Rgs17 |
| ENSMUSG00000105617 | 20.8728 | 3.32459 | 2.650372 | 0.000376 | 0.004978 | up | Gm43809 |
| ENSMUSG00000001018 | 31.452 | 64.9895 | -1.04705 | 0.000387 | 0.005093 | down | Snapin |
| ENSMUSG00000023224 | 1.77996 | 5.75517 | -1.69302 | 0.000389 | 0.005116 | down | Serping1 |
| ENSMUSG00000034613 | 2.25816 | 5.11902 | -1.18072 | 0.000414 | 0.005376 | down | Ppm1h |
| ENSMUSG00000036214 | 12.2082 | 4.73915 | 1.365148 | 0.000418 | 0.005431 | up | Znrd1as |
| ENSMUSG00000029304 | 838.383 | 1804.53 | -1.10594 | 0.000427 | 0.005521 | down | Spp1 ( Osteopontin) |
| ENSMUSG00000052713 | 2.87993 | 6.22567 | -1.1122 | 0.000428 | 0.005524 | down | Zfp608 |
| ENSMUSG00000107068 | 4.29006 | 11.3952 | -1.40935 | 0.000444 | 0.005693 | down | Gm42742 |
| ENSMUSG00000081375 | 9.00212 | 0.2903 | 4.954666 | 0.000448 | 0.005734 | up | Gm14686 |
| ENSMUSG00000078921 | 6.36909 | 14.5952 | -1.19633 | 0.000454 | 0.005773 | down | Tgtp2 |
| ENSMUSG00000066607 | 13.2242 | 29.4646 | -1.1558 | 0.000457 | 0.005805 | down | 6030419C18Rik |
| ENSMUSG00000097993 | 2.4372 | 8.27631 | -1.76376 | 0.000459 | 0.005823 | down | Ptprv |
| ENSMUSG00000043391 | 3.71769 | 7.98124 | -1.10221 | 0.000479 | 0.006023 | down | 2510009E07Rik |
| ENSMUSG00000020175 | 2.61005 | 7.10185 | -1.44412 | 0.000481 | 0.006047 | down | Rab36 |
| ENSMUSG00000069874 | 4.40187 | 9.9182 | -1.17196 | 0.000488 | 0.006117 | down | Irgm2 |
| ENSMUSG00000032727 | 9.54722 | 19.9087 | -1.06025 | 0.00049 | 0.006122 | down | Mier3 |
| ENSMUSG00000038876 | 33.0237 | 16.3185 | 1.016998 | 0.000512 | 0.006324 | up | Rnf146 |
| ENSMUSG00000053477 | 25.7127 | 53.699 | -1.06241 | 0.000522 | 0.006427 | down | Tcf4 |
| ENSMUSG00000021646 | 8.94356 | 19.7614 | -1.14376 | 0.000527 | 0.006468 | down | Mccc2 |
| ENSMUSG00000035208 | 5.09695 | 11.4613 | -1.16907 | 0.000545 | 0.006655 | down | Slfn8 |
| ENSMUSG00000044068 | 3.17466 | 7.2961 | -1.20052 | 0.000561 | 0.006813 | down | Zrsr1 |
| ENSMUSG00000026170 | 2.64959 | 6.76512 | -1.35234 | 0.000563 | 0.00683 | down | Cyp27a1 |
| ENSMUSG00000074682 | 3.33499 | 7.66057 | -1.19977 | 0.000566 | 0.006855 | down | Zcchc3 |
| ENSMUSG00000043004 | 1.68209 | 5.15107 | -1.61462 | 0.00057 | 0.006892 | down | Gng2 |
| ENSMUSG00000060572 | 4.3505 | 12.2171 | -1.48965 | 0.000571 | 0.006892 | down | Mfap2 |
| ENSMUSG00000025915 | 3.60516 | 8.83148 | -1.29259 | 0.00058 | 0.00699 | down | Sgk3 |
| ENSMUSG00000033152 | 2.37158 | 5.5809 | -1.23465 | 0.000585 | 0.007026 | down | Podxl2 |
| ENSMUSG00000068551 | 2.65627 | 5.98732 | -1.17251 | 0.000587 | 0.007045 | down | Zfp467 |
| ENSMUSG00000027536 | 2.85397 | 6.07508 | -1.08993 | 0.000594 | 0.007116 | down | Chmp4c |
| ENSMUSG00000036356 | 3.84814 | 9.80647 | -1.34957 | 0.000601 | 0.007185 | down | Csgalnact1 |
| ENSMUSG00000041729 | 2.72364 | 6.22969 | -1.19363 | 0.000618 | 0.007372 | down | Coro2b |
| ENSMUSG00000072955 | 0.8549 | 5.11799 | -2.58175 | 0.000668 | 0.007863 | down | Tmsb15l |
| ENSMUSG00000047238 | 5.72665 | 13.8202 | -1.27102 | 0.000671 | 0.007884 | down | Mageh1 |
| ENSMUSG00000006464 | 3.05757 | 6.59974 | -1.11002 | 0.000696 | 0.008105 | down | Bbs1 |
| ENSMUSG00000103081 | 0.10134 | 35.3006 | -8.44442 | 0.000718 | 0.008332 | down | Pcdhgb8 |
| ENSMUSG00000072620 | 4.91208 | 11.8181 | -1.2666 | 0.000727 | 0.008424 | down | Slfn2 |
| ENSMUSG00000072941 | 5.38547 | 11.6304 | -1.11076 | 0.000799 | 0.009137 | down | Sod3 |
| ENSMUSG00000073791 | 13.3903 | 6.08841 | 1.137052 | 0.000804 | 0.009174 | up | Efcab7 |
| ENSMUSG00000020592 | 65.113 | 133.353 | -1.03424 | 0.000831 | 0.00943 | down | Sdc1 |
| ENSMUSG00000042501 | 3.15488 | 8.02815 | -1.34748 | 0.000841 | 0.009502 | down | Cpa6 |
| ENSMUSG00000036882 | 3.74292 | 9.46957 | -1.33913 | 0.000863 | 0.00968 | down | Arhgap33 |
| ENSMUSG00000003031 | 23.6621 | 47.6069 | -1.00859 | 0.000898 | 0.010038 | down | Cdkn1b |
| ENSMUSG00000075273 | 2.89012 | 6.73154 | -1.21981 | 0.000942 | 0.010416 | down | Ttc30b |
| ENSMUSG00000020623 | 3.60806 | 8.1102 | -1.16852 | 0.000959 | 0.010528 | down | Map2k6 |
| ENSMUSG00000030231 | 24.0606 | 48.4473 | -1.00975 | 0.001022 | 0.011068 | down | Plekha5 |
| ENSMUSG00000060961 | 2.24628 | 5.60102 | -1.31815 | 0.001047 | 0.011282 | down | Slc4a4 |
| ENSMUSG00000038967 | 7.58635 | 15.797 | -1.05818 | 0.001119 | 0.011944 | down | Pdk2 |
| ENSMUSG00000056648 | 0.06413 | 6.8105 | -6.73055 | 0.001169 | 0.012358 | down | Hoxb8 |
| ENSMUSG00000021671 | 7.34868 | 15.5576 | -1.08207 | 0.001192 | 0.012557 | down | Poc5 |
| ENSMUSG00000016087 | 4.92464 | 10.2126 | -1.05226 | 0.001235 | 0.012933 | down | Fli1 |
| ENSMUSG00000042404 | 3.24568 | 8.1301 | -1.32475 | 0.001244 | 0.013005 | down | Dennd4b |
| ENSMUSG00000009470 | 30.5265 | 67.0259 | -1.13466 | 0.001246 | 0.013005 | down | Tnpo1 |
| ENSMUSG00000097697 | 5.00125 | 2.0238 | 1.305221 | 0.001258 | 0.013081 | up | 4833412C05Rik |
| ENSMUSG00000031129 | 2.76778 | 6.25093 | -1.17534 | 0.001281 | 0.013265 | down | Slc9a9 |
| ENSMUSG00000006611 | 4.1246 | 10.545 | -1.35424 | 0.001294 | 0.013393 | down | Hfe |
| ENSMUSG00000032291 | 0.77366 | 5.12901 | -2.72891 | 0.001309 | 0.013517 | down | Crabp1 |
| ENSMUSG00000035142 | 3.34346 | 8.16594 | -1.28828 | 0.001311 | 0.013526 | down | Nubpl |
| ENSMUSG00000025207 | 2.26176 | 5.10169 | -1.17353 | 0.001374 | 0.01405 | down | Sema4g |
| ENSMUSG00000064361 | 2.69954 | 30.7522 | -3.5099 | 0.001474 | 0.014861 | down | mt-Tr |
| ENSMUSG00000042675 | 13.392 | 32.3493 | -1.27236 | 0.00151 | 0.015173 | down | Ypel3 |
| ENSMUSG00000021240 | 5.93648 | 12.4614 | -1.06979 | 0.001558 | 0.015552 | down | Abcd4 |
| ENSMUSG00000051177 | 2.16461 | 5.03088 | -1.2167 | 0.001657 | 0.016338 | down | Plcb1 |
| ENSMUSG00000097141 | 5.42124 | 11.6881 | -1.10834 | 0.001666 | 0.016401 | down | Gm10524 |
| ENSMUSG00000048355 | 3.11628 | 8.17466 | -1.39133 | 0.00169 | 0.016583 | down | Arxes1 |
| ENSMUSG00000037395 | 12.8397 | 25.9422 | -1.01468 | 0.001707 | 0.016673 | down | Rcor3 |
| ENSMUSG00000038527 | 4.69978 | 9.73105 | -1.05 | 0.001753 | 0.016995 | down | C1rl |
| ENSMUSG00000038451 | 6.17265 | 20.3275 | -1.71947 | 0.001768 | 0.017097 | down | Spsb2 |
| ENSMUSG00000024885 | 9.35877 | 20.4296 | -1.12627 | 0.001826 | 0.017478 | down | Aldh3b1 |
| ENSMUSG00000062040 | 2.14101 | 5.04178 | -1.23564 | 0.001883 | 0.017887 | down | Zfp27 |
| ENSMUSG00000093219 | 28.4542 | 0.48799 | 5.865635 | 0.001906 | 0.01804 | up | Mir3113 |
| ENSMUSG00000056498 | 3.16369 | 6.82924 | -1.11012 | 0.001911 | 0.018058 | down | Tmem154 |
| ENSMUSG00000048106 | 4.848 | 10.202 | -1.07339 | 0.00193 | 0.018157 | down | 4632415L05Rik |
| ENSMUSG00000042349 | 8.20307 | 17.6801 | -1.10789 | 0.00203 | 0.018918 | down | Ikbke |
| ENSMUSG00000030037 | 10.4909 | 5.16253 | 1.022991 | 0.002059 | 0.019132 | up | Mrpl53 |
| ENSMUSG00000053656 | 12.6026 | 6.15453 | 1.033998 | 0.002112 | 0.019516 | up | Dancr |
| ENSMUSG00000038793 | 2.14013 | 5.87295 | -1.45639 | 0.002121 | 0.019557 | down | Lefty1 |
| ENSMUSG00000039976 | 2.26226 | 5.21032 | -1.2036 | 0.00214 | 0.019675 | down | Tbc1d16 |
| ENSMUSG00000027695 | 4.72973 | 10.3771 | -1.13358 | 0.002148 | 0.01972 | down | Pld1 |
| ENSMUSG00000040187 | 6.29948 | 13.7118 | -1.12211 | 0.002148 | 0.01972 | down | Arntl2 |
| ENSMUSG00000062309 | 5.35205 | 13.3606 | -1.31982 | 0.002271 | 0.020583 | down | Rpp25 |
| ENSMUSG00000062515 | 8.5231 | 19.5948 | -1.20102 | 0.002377 | 0.021383 | down | Fabp4 |
| ENSMUSG00000078867 | 5.10997 | 2.48136 | 1.042187 | 0.002394 | 0.021481 | up | Gm14418 |
| ENSMUSG00000106951 | 2.81034 | 6.58357 | -1.22812 | 0.002508 | 0.022151 | down | 5930430L01Rik |
| ENSMUSG00000026239 | 67.6665 | 158.005 | -1.22346 | 0.002511 | 0.022171 | down | Pde6d |
| ENSMUSG00000035513 | 1.99028 | 5.25723 | -1.40133 | 0.00254 | 0.022357 | down | Ntng2 |
| ENSMUSG00000110104 | 109.393 | 0.00183 | 15.86692 | 0.00261 | 0.022885 | up | Gm45717 |
| ENSMUSG00000046312 | 3.53797 | 7.08134 | -1.0011 | 0.002653 | 0.023213 | down | AI464131 |
| ENSMUSG00000029869 | 4.62899 | 9.34399 | -1.01334 | 0.002667 | 0.023287 | down | Ephb6 |
| ENSMUSG00000033276 | 2.64878 | 6.37418 | -1.26691 | 0.00272 | 0.023668 | down | Stk36 |
| ENSMUSG00000024511 | 4.12021 | 9.14771 | -1.15069 | 0.002731 | 0.023717 | down | Rab27b |
| ENSMUSG00000031785 | 5.15299 | 12.2325 | -1.24724 | 0.002736 | 0.02374 | down | Adgrg1 |
| ENSMUSG00000035357 | 5.06869 | 10.3264 | -1.02665 | 0.00291 | 0.024856 | down | Pdzrn3 |
| ENSMUSG00000004791 | 10.6204 | 22.5937 | -1.08908 | 0.002966 | 0.025205 | down | Pgf |
| ENSMUSG00000059970 | 5.02861 | 10.6611 | -1.08412 | 0.003002 | 0.025451 | down | Hspa2 |
| ENSMUSG00000090115 | 3.95629 | 7.94179 | -1.00532 | 0.003009 | 0.025495 | down | Usp49 |
| ENSMUSG00000047694 | 8.04568 | 18.0382 | -1.16477 | 0.003067 | 0.025816 | down | Yipf6 |
| ENSMUSG00000103009 | 4.11087 | 9.04299 | -1.13736 | 0.003197 | 0.026742 | down | Gm4430 |
| ENSMUSG00000078141 | 6.26386 | 23.7109 | -1.92043 | 0.00328 | 0.027299 | down | Gm2399 |
| ENSMUSG00000035773 | 7.8511 | 3.22688 | 1.282756 | 0.003362 | 0.027805 | up | Kiss1r |
| ENSMUSG00000045679 | 13.0609 | 26.7236 | -1.03286 | 0.003373 | 0.027888 | down | Pqlc3 |
| ENSMUSG00000021238 | 8.16083 | 16.8169 | -1.04312 | 0.003395 | 0.028043 | down | Aldh6a1 |
| ENSMUSG00000007646 | 4.30542 | 8.66837 | -1.0096 | 0.003459 | 0.028454 | down | Rad51c |
| ENSMUSG00000078716 | 2.17421 | 5.78151 | -1.41095 | 0.003547 | 0.029043 | down | Tmem8b |
| ENSMUSG00000040998 | 5.84825 | 13.3304 | -1.18864 | 0.003694 | 0.029954 | down | Npnt |
| ENSMUSG00000060224 | 3.80294 | 7.73105 | -1.02355 | 0.003748 | 0.030353 | down | Pyroxd2 |
| ENSMUSG00000003411 | 26.4004 | 67.449 | -1.35324 | 0.003784 | 0.030538 | down | Rab3b |
| ENSMUSG00000042320 | 2.21272 | 5.93616 | -1.42371 | 0.003954 | 0.031661 | down | Prox2 |
| ENSMUSG00000048277 | 31.9632 | 92.4002 | -1.53149 | 0.004033 | 0.032172 | down | Syngr2 |
| ENSMUSG00000020160 | 4.68364 | 9.48559 | -1.01811 | 0.004066 | 0.032356 | down | Meis1 |
| ENSMUSG00000017830 | 7.26281 | 15.9203 | -1.13227 | 0.004165 | 0.032978 | down | Dhx58 |
| ENSMUSG00000026200 | 6.61302 | 14.2611 | -1.10871 | 0.004208 | 0.033241 | down | Glb1l |
| ENSMUSG00000112478 | 8.66014 | 17.5776 | -1.02127 | 0.004384 | 0.034346 | down | AC153370.2 |
| ENSMUSG00000031586 | 11.1281 | 23.0911 | -1.05314 | 0.004587 | 0.035444 | down | Rbpms |
| ENSMUSG00000035967 | 5.38097 | 11.2441 | -1.06324 | 0.004714 | 0.036149 | down | Ints6l |
| ENSMUSG00000028943 | 5.15063 | 2.0071 | 1.359638 | 0.00479 | 0.03661 | up | Espn |
| ENSMUSG00000043333 | 5.67316 | 12.7636 | -1.16981 | 0.004892 | 0.037272 | down | Rhbdl2 |
| ENSMUSG00000107881 | 9.12118 | 19.2655 | -1.07873 | 0.005084 | 0.038273 | down | Gm44250 |
| ENSMUSG00000045868 | 2.81073 | 6.66259 | -1.24514 | 0.005292 | 0.039389 | down | Gvin1 |
| ENSMUSG00000087143 | 1.90629 | 7.35852 | -1.94865 | 0.0053 | 0.039435 | down | A830082K12Rik |
| ENSMUSG00000043556 | 1.81379 | 5.06444 | -1.4814 | 0.005419 | 0.040193 | down | Fbxl7 |
| ENSMUSG00000021469 | 3.72165 | 7.75906 | -1.05994 | 0.005755 | 0.042155 | down | Msx2 |
| ENSMUSG00000030170 | 5.73116 | 11.6774 | -1.02682 | 0.005863 | 0.04274 | down | Wnt5b |
| ENSMUSG00000105954 | 2.90427 | 9.16428 | -1.65785 | 0.005939 | 0.043018 | down | Gm42793 |
| ENSMUSG00000103332 | 0.30009 | 17.6548 | -5.87854 | 0.006104 | 0.043898 | down | Pcdhga2 |
| ENSMUSG00000076432 | 169.034 | 346.484 | -1.03547 | 0.006274 | 0.044746 | down | Ywhaq |
| ENSMUSG00000028539 | 2.02722 | 5.87301 | -1.5346 | 0.006295 | 0.044839 | down | Artn |
| ENSMUSG00000097649 | 2.48563 | 5.97056 | -1.26426 | 0.006359 | 0.04517 | down | Gm10561 |
| ENSMUSG00000006575 | 7.89263 | 3.74614 | 1.075102 | 0.006435 | 0.045552 | up | Rundc3a |
| ENSMUSG00000021709 | 27.7485 | 57.4107 | -1.04891 | 0.00649 | 0.04588 | down | Erbin |
| ENSMUSG00000026988 | 14.0005 | 28.2337 | -1.01194 | 0.006678 | 0.046855 | down | Wdsub1 |
| ENSMUSG00000024678 | 0.15522 | 7.3916 | -5.5735 | 0.006701 | 0.046964 | down | Ms4a4d |
| ENSMUSG00000034947 | 15.0856 | 35.351 | -1.22858 | 0.00694 | 0.048375 | down | Tmem106a |
| ENSMUSG00000000782 | 7.99559 | 17.2561 | -1.10983 | 0.006993 | 0.048609 | down | Tcf7 |
| ENSMUSG00000025255 | 7.3936 | 15.3305 | -1.05205 | 0.007065 | 0.04895 | down | Zfhx4 |
| ENSMUSG00000025176 | 3.27534 | 7.64618 | -1.22309 | 0.007151 | 0.049381 | down | Hoga1 |

References:

1. Huang, S.; Xu, L. L.; Sun, Y. X.; Wu, T. Y.; Wang, K. X.; Li, G., An improved protocol for isolation and culture of mesenchymal stem cells from mouse bone marrow. *J Orthop Transl* **2015,** *3* (1), 26-33.

2. Lin, W.; Xu, L.; Pan, Q.; Lin, S.; Feng, L.; Wang, B.; Chen, S.; Li, Y.; Wang, H.; Li, Y.; Wang, Y.; Lee, W. Y. W.; Sun, D.; Li, G., Lgr5-overexpressing mesenchymal stem cells augment fracture healing through regulation of Wnt/ERK signaling pathways and mitochondrial dynamics. *FASEB J* **2019,** *33* (7), 8565-8577.

3. Xia, W. F.; Tang, F. L.; Xiong, L.; Xiong, S.; Jung, J. U.; Lee, D. H.; Li, X. S.; Feng, X.; Mei, L.; Xiong, W. C., Vps35 loss promotes hyperresorptive osteoclastogenesis and osteoporosis via sustained RANKL signaling. *The Journal of cell biology* **2013,** *200* (6), 821-37.
